# Green Synthesis of Gold Nanoparticles Using Liquiritin and Other Phenolics from *Glycyrrhiza glabra* and Their Anti-Inflammatory Activity

**DOI:** 10.3390/jfb15040095

**Published:** 2024-04-06

**Authors:** Ali O. E. Eltahir, Kim L. Lategan, Oladipupo M. David, Edmund J. Pool, Robert C. Luckay, Ahmed A. Hussein

**Affiliations:** 1 Chemistry Department, Cape Peninsula University of Technology, Bellville 7535, South Africa; aliomers250@gmail.com; 2 Department of Medical Bioscience, University of Western the Cape, Bellville 7535, South Africa; klategan@uwc.ac.za (K.L.L.); 3681075@myuwc.ac.za (O.M.D.); epool@uwc.ac.za (E.J.P.); 3 Department of Chemistry and Polymer Science, Stellenbosch University, Matieland, Stellenbosch 7602, South Africa; rcluckay@sun.ac.za

**Keywords:** *Glycyrrhiza glabra*, licorice, liquiritin, phenolic compounds, gold nanoparticles, lipopolysaccharide, cell viability, anti-inflammatory

## Abstract

Phenolic compounds are the main phytochemical constituents of many higher plants. They play an important role in synthesizing metal nanoparticles using green technology due to their ability to reduce metal salts and stabilize them through physical interaction/conjugation to the metal surface. Six pure phenolic compounds were isolated from licorice (*Glycyrrhiza glabra*) and employed in synthesizing gold nanoparticles (AuNPs). The isolated compounds were identified as liquiritin (**1**), isoliquiritin (**2**), neoisoliquiritin (**3**), isoliquiritin apioside (**4**), liquiritin apioside (**5**), and glabridin (**6**). The synthesized AuNPs were characterized using UV, zeta sizer, HRTEM, and IR and tested for their stability in different biological media. The phenolic isolates and their corresponding synthesized NP conjugates were tested for their potential in vitro cytotoxicity. The anti-inflammatory effects were investigated in both normal and inflammation-induced settings, where inflammatory biomarkers were stimulated using lipopolysaccharides (LPSs) in the RAW 264.7 macrophage cell line. LPS, functioning as a mitogen, promotes cell growth by reducing apoptosis, potentially contributing to observed outcomes. Results indicated that all six pure phenolic isolates inhibited cell proliferation. The AuNP conjugates of all the phenolic isolates, except liquiritin apioside (**5**), inhibited cell viability. LPS initiates inflammatory markers by binding to cell receptors and setting off a cascade of events leading to inflammation. All the pure phenolic isolates, except isoliquiritin, neoisoliquiritin, and isoliquiritin apioside inhibited the inflammatory activity of RAW cells in vitro.

## 1. Introduction

Various chemical and physical syntheses of nanoparticles have been applied for the biosynthesis of AuNPs [1,2]. Physicochemical properties can have substantial effects, such as target-binding activities, and increase tolerance toward biocompatibility [3]. The combination of a high specific surface area, safety, biocompatibility, and the ability to easily modify their surfaces, along with gold’s strong affinity for sulfur atoms and its remarkable cell and tissue penetration capabilities, position AuNPs as a superb platform for diagnostics and therapeutics in the field of biomedicine. Furthermore, incorporating well-defined capping agents with a safety margin and established pharmacological profiles has the potential to expand the significance of AuNP/natural product conjugates, creating a novel and valuable platform for addressing inflammation [4,5,6,7,8].

A fundamental understanding of the nanoparticle’s surface and the capping agents’ nature is crucial for designing and developing well-characterized nanomaterials [9].

Green synthesis, in line with green chemistry principles, provides many advantages. One of these benefits is the utilization of abundant, easily accessible, and affordable biodegradable natural sources that serve as efficient reducing and stabilizing agents. Furthermore, eco-friendly solvents are frequently employed, safeguarding the environment against detrimental chemical residues. Moreover, the nanoparticles produced through green synthesis demonstrate considerably reduced toxicity levels [10,11].

Particular interest has been directed towards using natural products as reducing and capping agents [12], and AuNPs capped with different phenolic compounds such as epigallocatechin gallate (EGCG) [13], mangiferin [14], quercetin [15], proanthocyanidin dimer [16], hypoxoside [17,18], hesperidin [19,20], and tricetin have been reported [21]. Capping agents, in most cases, are responsible not only for the stability of the NPs but also for enhancing their activity and selectivity [22,23]. Due to the chemical nature of the phenolic compounds, they can form, stabilize, and activate the AuNPs. Phenolic compounds are widely distributed in nature and have essential biological functions in plants [24,25]. Additionally, they have a wide range of pharmacological properties and are beneficial for human health [25,26,27].

Licorice (*Glycyrrhiza glabra*) is a known medicinal plant that has a wide range of traditional and pharmacological activities. The chemistry of the plant has been studied extensively and several types of flavonoids and triterpenoid glycosides have been shown [28]. Different biological activities, including the anti-inflammatory activity of the total extract and some isolated compounds, have been reported in vivo and in vitro [29,30].

The future application of metal NPs as biological modulators in treating various human pathologies depends on the presence of both safe and bioactive conjugates with well-defined structures. Fulfilling these requirements entails employing green technology for safety purposes and utilizing active capping agents that are singular and well-defined, moving away from the use of total extracts. Continuing our research endeavors, we strive to employ well-defined metal NP conjugates with medicinal applications [17,18,31]. This study presents the green synthesis and characterization of six newly synthesized AuNPs conjugated with pure phenolics isolated from licorice for the first time. Additionally, we investigate their potential as inhibitors of inflammatory biomarkers.

Inflammation is one of the immune defense mechanisms of organisms when their cells are adversely affected by microbial, physical, or chemical injury [32]. Although inflammation is typically regarded as a vertebrate response to adverse effects, studies have shown that inflammation also occurs in some invertebrates [33]. The adversely affected cells send out chemical messengers, namely inflammatory chemicals and cytokines, to inform the immune system and other cells of the adverse event/s. Several in vitro and in vivo inflammatory messengers or biomarkers have been well characterized, and these include nitric oxide (NO) and the cytokines interleukin 6 (IL-6) and interferon gamma (IFN-γ) [34].

This study is limited to phenolic compounds from licorice and their ability to inhibit inflammatory biomarkers synthesized by the RAW 264.7 macrophage cell line. Previous studies by us and others [35,36,37] showed that addition of lipopolysaccharides (LPSs) to RAW 264.7 culture medium induced several biomarkers of inflammation, such as nitric oxide, interleukin 6, and tumor necrosis factor. LPSs’ addition to cultures also resulted in the upregulation of inducible nitric oxide synthase (iNOS) [38,39].

NO is an important signaling molecule that plays a role in initializing the inflammatory process. NO formation is catalyzed by nitric oxide synthases (NOSs), as these enzymes convert arginine into citrulline via the co-factors oxygen and nicotinamide adenine dinucleotide phosphate (NADPH), with NO being produced during this process. There are three isoforms of NOS, which can be found in a number of tissue and cell types. The three isoforms of NOS are neuronal NOS (nNOS), endothelial NOS (eNOS), and inducible NOS (iNOS) [40]. iNOS is induced via LPS stimulation as LPS acts via the CD14 receptor on the cell membrane and subsequently activates NFκβ, leading to the expression of iNOS and subsequent NO production [41]. 

## 2. Materials and Methods

### 2.1. Materials 

#### 2.1.1. Chemicals and Reagents

All solvents used for extraction and column chromatography were general-purpose reagents. Methanol (AR), hexane, dichloromethane, methanol (HPLC grade), ethanol, ethyl acetate, sodium tetrachloroaurate (III) dihydrate (NaAuCl_4_·2H_2_O, 99.99%), and glycine (Gly) were purchased from Sigma-Aldrich (Cape Town, South Africa). Polyethylene glycol (PEG), cysteine (Cys), Dulbecco’s Modified Eagle (DMEM), and bovine serum albumin (BSA) from Miles Laboratories (Pittsburgh, PA, USA), and *N*-acetyl-L-cysteine from Boehringer Mannheim GmbH (Mannheim, Germany). Polystyrene 96-well microtiter plates were obtained from Greiner Bio-one GmbH (Frickenhausen, BY, Germany). Silica gel 60 (0.063–0.200 mm) and Sephadex (LH-20) were supplied by Merck (Darmstadt, Germany). The murine macrophage cell line, RAW 264.7, was obtained from the American Type Culture Collection (ATCC TIB-71, Manassas, VA, USA).

#### 2.1.2. Equipment 

A SPECTROstar Nano (BMG LABTECH, Ortenberg, Germany) 2450 ultraviolet/visible (UV-Vis) spectrophotometer was used to monitor the characteristic peaks of the AuNPs. High-resolution transmission electron microscopy (HRTEM) (FEI Tecnai G2 F20 S-Twin HR-TEM, Hillsboro, OR, USA, operated at 200 kV) was used to study the morphology of the AuNPs. An Oxford energy-dispersive X-ray spectroscopy system inside a Zeiss Auriga Field Emission Scanning Electron Microscope (Miramar, Oxfordshire, UK) was used for elemental analysis. Dynamic light scattering (DLS) analysis was carried out using a Malvern Zetasizer (Malvern Ltd., Malvern, UK) at 25 °C and a 90° angle. A Fourier transform infrared (FTIR) spectrophotometer (Waltham, MA, USA) was used to measure the IR spectra. The 1D NMR (^1^H, ^13^C and DEPT-135) and 2D spectra were measured using a Bruker spectrometer (Rheinstetten, Germany) operating at 400 MHz (for ^1^H) and 100 MHz (for ^13^C).

### 2.2. Methods

#### 2.2.1. Extraction and Purification of Phenolic Compounds 

Licorice powder (500.0 g) was extracted with methanol at 60 °C (3 L × 2 h × 2 times). After concentration, it yielded 104.3 g. Fifty grams of the total extract (TE) was applied to the silica gel column and eluted with hexane/ethyl acetate gradient of increasing polarity (starting from EtOAc 0% (2L), 10% (2L), 30% (2L), 50% (2L), 80% (2L), and 100 (1L)).

**Scheme 1:**
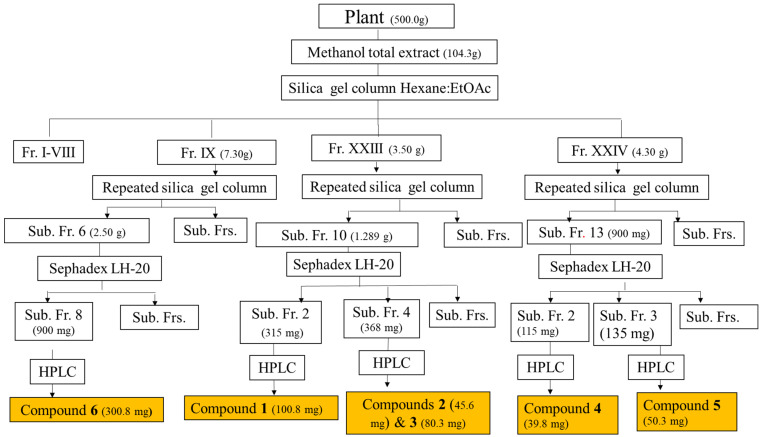
Schematic diagram for the isolation and purification of the major compounds from licorice, Fr(s). = Fraction(s).

Collected fractions were pooled according to their profiles on the thin layer chromatography (TLC) to afford 24 major fractions coded as I to XXIV. Specific fractions were selected (according to their ability to synthesize NPs) and rechromatographed to yield six major compounds as follows (Scheme 1): 

The main fraction XXIII (3.50 g) was chromatographed on silica gel using a gradient of hexane and EtOAc (80:20 to 0:100). The obtained sub-fraction 10 was purified on Sephadex (Merck, Cape Town, South Africa) using isocratic 80% aqueous ethanol then semi-prep HPLC using a gradient of MeOH and DIW (de-ionized water) (40:60 to 60:80 in 30 min, and to 80:90 in 10 min, then to 100% MeOH in 10 min) to give compounds **1** (100.8 mg), **2** (45.6 mg), and **3** (80.3 mg).

The main fraction XXIV (4.30 g) was chromatographed on silica gel. Subfractions 2 and 3 were chromatographed separately on Sephadex and semi-prep HPLC, as previously mentioned, to give compounds **4** (39.8 mg) and **5** (50.3 mg).

The main fraction IX (7.30 g) was chromatographed on silica gel using a gradient of hexane and EtOAc (80:20 to 0:100), and the sub-fraction 6 was purified on Sephadex and then semi-prep HPLC using a mixture of MeOH and DIW (1:1, isocratic) to give compound **6** (300.8 mg). The analysis of the total extract using LC-MS was carried out to profile and identify the compounds associated with nanoparticles when the total extract was used (Supplementary Material Figure S1).

#### 2.2.2. Green Synthesis of Gold Nanoparticles

Total extracts/pure compounds were used to prepare AuNPs: a fresh 16.0 mg/mL stock solution of each extract/pure compound in 50% methanol/distilled water was prepared. Serial dilutions from 2.66 to 0.083 mg/mL were prepared in a 96-well plate. To 50 μL of the TE/compounds solution, 250 μL of 0.00125 mM of gold salt was added, and plates were incubated at 60 °C. The change of color from light yellow to reddish or purple indicated the successful formation of AuNPs [42]. The optimum concentrations were selected to scale up the AuNPs’ preparation to obtain enough material for biological studies. The formation of the AuNPs was further confirmed by recording a UV–vis spectrum in the 300–800 nm range [43].

#### 2.2.3. Characterization of Gold Nanoparticles

Different characterization techniques such as UV–vis, HRTEM, FTIR, and DLS were used to investigate the formation of AuNPs and their various physicochemical properties. The absorption bands due to electrons confined on the surface were measured using a microtitre plate reader (BMG Labtech, Ortenberg, Germany). Zeta potential, hydrodynamic size, and polydispersity indices (Pdis) were measured using Malvern Zetasizer. HRTEM images were analyzed using ImageJ software, 1.50b version 1.8.0_60 (http://imagej.nih.gov/ij (accessed on 17 May 2022)) and Origin pro-2022b (64 bits) software, respectively. Vibrational bands of possible functional groups of the synthesized AuNPs were recorded on FTIR at a 400−4000 cm^−1^ transmission mode.

#### 2.2.4. Stability Study

The method described by Elbagory et al. in 2016 was utilized [31]. Briefly, 100 µL of the AuNP solutions were incubated with equal volume (1:1) of the buffer solutions BSA, PBS, PEG, Gly, and Cys) in a 96-well plate. The final concentrations of the biological media in the final mixture were as follows: 5% PEG, 0.5% cysteine, glycine, PBS, and 0.5% BSA. The stability of the AuNPs was evaluated by measuring the changes in the UV–vis spectra after 24 h.

#### 2.2.5. Biological Study

##### Preparation of Stock Solutions

AuNP conjugates, total methanolic extract, and pure compounds were reconstituted in DMSO. After that, a 1.2 mg/mL stock solution of the relevant treatment (AuNP conjugates, total methanolic extract, and pure compounds) was prepared in sterile distilled water. Before cell exposure, the stock solutions were briefly sonicated (QSonica, LLC. Misonixsonicators, XL-200 Series, Newtown, CT, USA) on ice for approximately 3 min. After that, the stock solutions were further diluted in complete Dulbecco’s Modified Eagle’s Medium (DMEM, Lonza, Cape Town, South Africa). Complete DMEM consisted of 10% heat-inactivated fetal bovine serum (FBS, Life Technologies ThermoFisher Scientific, Bedford, MA, USA), 1% antibiotic/antimycotic solution, 1% glutamax, and 0.5% gentamicin (Sigma-Aldrich, St. Louis, MO, USA).

##### Cell Culture and Exposure

Cells were cultured in complete DMEM, incubated at 37 °C in a humidified atmosphere of 5% CO_2_, and sub-cultured every 2–3 days.

The RAW 264.7 cells were seeded at 1 × 10^6^ cells/mL in 96-well tissue culture-treated plates for cell viability and nitric oxide assays. The seeded cells were incubated under standard tissue culture conditions at 37 °C in a humidified atmosphere of 5% CO_2_ for approximately 24 h until the cells reached 70–80% confluence. The cells were subsequently exposed to the relevant AuNP conjugates, total extract, or compounds. After that, the cells were either left unstimulated or stimulated with 1 μg/mL lipopolysaccharide (LPS, from *E. coli* O111:B4, Sigma-Aldrich) to yield a final concentration range of 0–100 μg/mL of the appropriate treatment. The cells were then incubated overnight under standard tissue culture conditions.

##### Cell Viability Assay

The supernatants were removed after incubation, and cells were washed with phosphate-buffered saline (PBS, Lonza). Cell viability was assessed by adding 50 μL of a 1/10 dilution of 2-(4-iodophenyl)-3-(4-nitrophenyl)-5-(2,4-disulfophenyl)-2*H*-tetrazolium (WST-1, Roche, Basel, Switzerland) in complete DMEM medium to each well. Metabolically active cells convert the WST-1 reagent to a formazan that can be measured spectrophotometrically. Formazan formation was determined by reading the plate at 450 nm immediately after adding WST-1 and again after an incubation period of 1 h at 37 °C. The increase in absorbance is proportional to formazan formation and directly proportional to cell viability. The difference between the zero-absorbance reading and 1 hr incubation was used to calculate the percentage of cell viability. Cell viability was calculated as a percentage of the control using the below formula:

(1) $$\mathrm{Cell}\;\mathrm{viability}\;\mathrm{as}\;a\;\%\;\mathrm{of}\;\mathrm{Control}=\frac{T1-T0}{U1-U0}\times 100$$

where T1 is the absorbance of the treated sample after 1 h, T0 is the absorbance of the treated at 0 h, U1 is the absorbance of the untreated sample after 1h, and U0 is the absorbance of the untreated sample at 0 h.

##### Nitric Oxide (NO) Assay

In the initial development of the assay, polymyxin inhibition of LPS inflammatory activity was checked. Polymyxin was established to inhibit LPS-induced inflammatory activity in macrophage cultures [37]. Cell culture supernatants were removed after the overnight incubation with the relevant treatment. They were used to determine the amount of nitrite produced by the cells and used as an indication of NO production. The amount of NO produced was determined against a 0–100 μM nitrite standard range (Sigma-Aldrich). Culture supernatants or nitrite standard (50 μL) were mixed with 50 μL of Griess reagent (Sigma-Aldrich). The absorbance was subsequently read spectrophotometrically at 540 nm (Multiskan Ex, Thermo Electron Corporation, Vantaa, Finland), and the amount of NO produced by the cells was quantified. NO levels were not determined at cytotoxic concentrations. 

### 2.3. Statistical Analysis

All experiments were performed in triplicate, and the data were calculated using Microsoft Excel. Data are represented as the mean ± standard error of the mean (SEM). Statistical differences with *p* < 0.001 were deemed significant after conducting a one-way analysis of variance (ANOVA) using Sigma Plot 12.0 (Systat Software Inc., San Jose, CA, USA).

## 3. Results 

### 3.1. Chemical Characterization of the Isolated Compounds 

**Figure 1:**
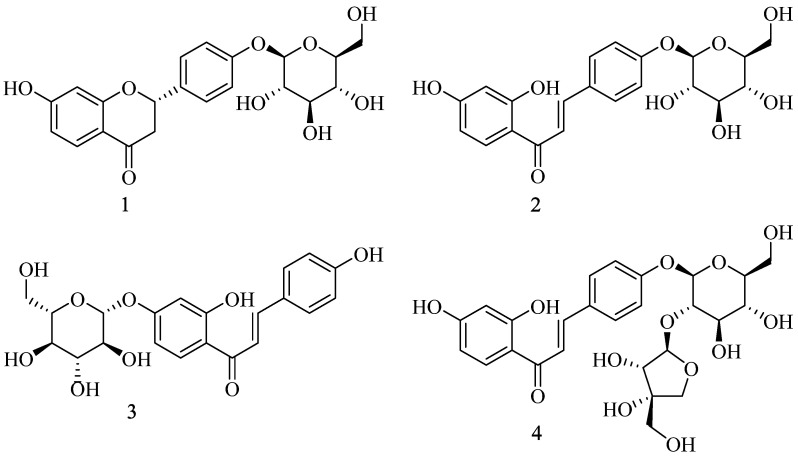

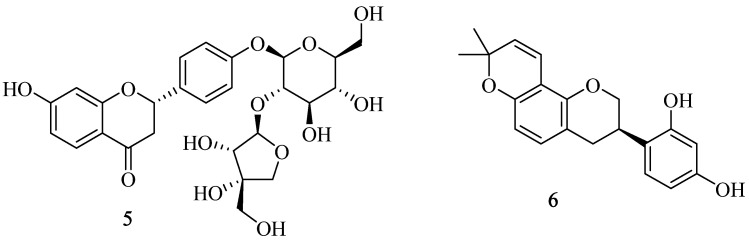
The chemical structures of the isolated compounds (**1**–**6**) from licorice.

Chromatographic purification of the total extract using different techniques, including HPLC (Scheme 1), resulted in the isolation of six pure major compounds, namely, liquiritin (**1**), isoliquiritin (**2**), neoisoliquiritin (**3**), isoliquiritin apioside (**4**) [44], liquiritin apioside (**5**), and glabridin (**6**) [45]. The compounds (Figure 1) were identified based on detailed spectroscopic analysis (Figure S2.1–S2.6 and Table 1) and comparison with the reported data [46].

### 3.2. Preparation and Characterization of AuNPs

At the outset, the total extract exhibited promising potential for forming AuNPs. Subsequently, through a phytochemical process, we isolated six major compounds with significant reducing capabilities, which were then utilized for the green synthesis of AuNPs. The synthesized nanoparticles underwent characterization using various spectroscopic techniques, including ultraviolet–visible (UV–vis) (see Figure S3 and Table 2) [13], particle size and zeta potential (Zp) measurement (see Table 2 and Figure S4), high-resolution transmission electron microscopy (HRTEM) (see Table 2 and Figure S5), X-ray diffraction (XRD) [47] (see Figure S8 and Table 2), and Fourier transform infrared (FTIR) spectroscopy [43] (see Figures S9 and S10).

### 3.3. Stability Study

The stability of nanoparticles in biological media has different durations depending on the type of nanoparticle. The stability study was carried out in various media, including bovine serum albumin (BSA), phosphate-buffered saline (PBS), polyethylene glycol (PEG), glycine (Gly), and cysteine (Cys). The media were added (1:1, *v*/*v*) to the synthesized AuNPs. These were placed in a 96-well plate and incubated at 37 °C for different periods. UV–vis monitored the stability for a period of 24 h (Figures S6 and S7). 

### 3.4. Biological Activity of AuNPs

Inflammation is a host response to harmful stimuli and features traditional signs such as redness, swelling, heat, and pain. This study aimed to assess the activity of the crude extract, pure compounds, and their nanoparticles on NO production [29,30].

#### 3.4.1. Cell Viability

RAW cell viability in the presence of the extract, compounds, and nanoparticles was determined using the WST-1 assay in unstimulated and LPS-stimulated conditions (Table 3). Cell viability indicates whether a specific additive to cultured cells activates or reduces the number of viable cells and usually is indirectly proportional to the toxicity of the additive.

#### 3.4.2. Liquiritin (**1**) and Liquiritin Conjugated to AuNPs (**1**@AuNPs)

**Figure 2:**
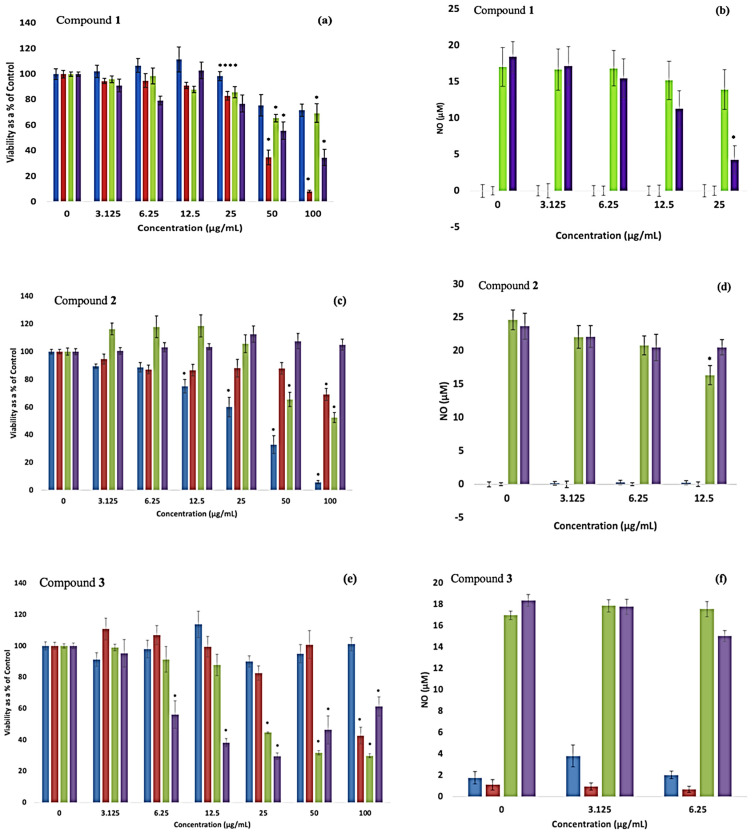

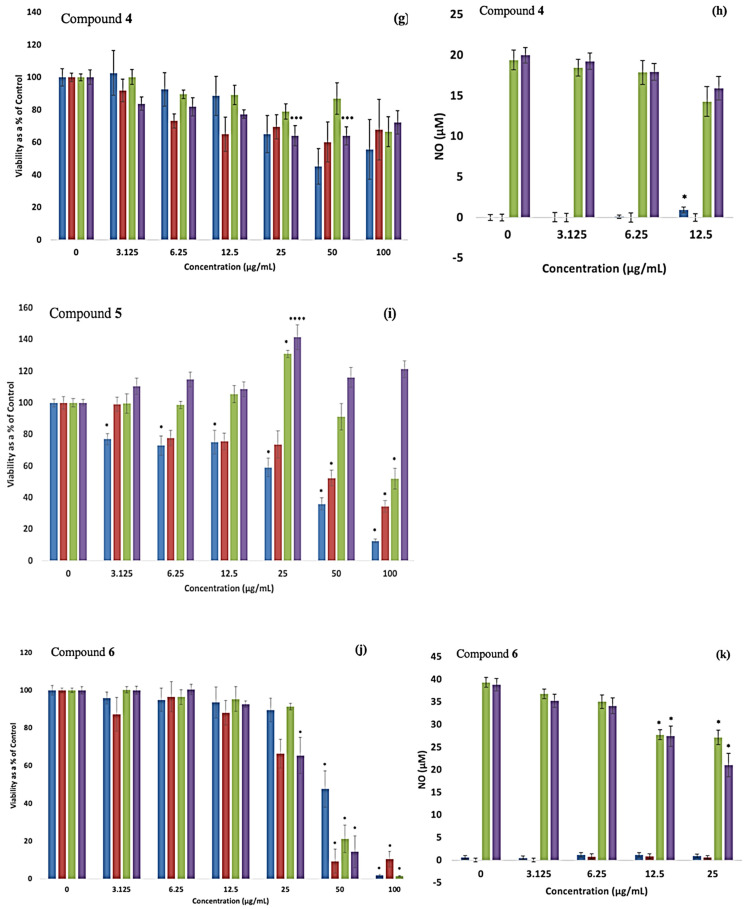

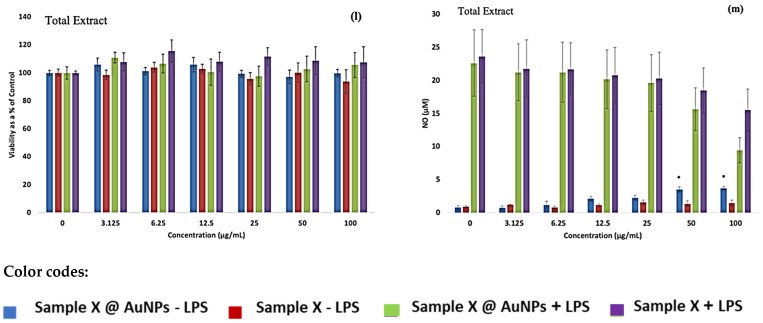
The assessment of RAW 264.7 cell viability and NO after 24 h exposure to various AuNPs, TE, and compounds in either the presence or absence of LPS. Cells were treated with the following: (**a**,**b**) compound **1**; (**c**,**d**) compound **2**; (**e**,**f**) compound **3**; (**g**,**h**) compound **4**; (**i**) compound **5**; (**j**,**k**) compound **6**; (**l**,**m**) TE. Data are presented as mean ± SEM, where the *p*-value indicates statistical significance against the relevant control, using one-way ANOVA. * *p* < 0.001, *** *p* = 0.003, and **** *p* = 0.005. Sample X + LPS and green should be Sample X + AuNPs + LPS.

Under a simulated inflammatory response, RAW cells experienced a significant (*p* < 0.001) loss of viability at 50 μg/mL AuNPs (Figure 2a). In comparison, compound **1** was cytotoxic at concentrations of 50–100 μg/mL in the absence and presence of LPS, with approximate IC_50_ values of 39.7 ± 1.6 and 60.5 ± 4.5 μg/mL, respectively (Table 3).

#### 3.4.3. Isoliquiritin (**2**) and **2**@AuNPs

The AuNPs in the absence of LPS significantly (*p* < 0.001) decreased RAW cell viability at concentrations ≥ 12.5 μg/mL (Figure 2c), with the cell viability ranging from 75−5%, (IC_50_ ~ 29.2 ± 2.3 μg/mL) (Table 2). However, a mitogenic effect was seen in the presence of LPS as the AuNPs induced significant (*p* < 0.001) cytotoxicity only at concentrations of 50 μg/mL, ranging from 65−8% viability. A noticeable (*p* < 0.001) drop in viability was seen only at 100 μg/mL of compound **2** under basal conditions, and no cytotoxicity was exhibited under a simulated inflammatory response.

#### 3.4.4. Neoisoliquiritin (**3**) and **3**@AuNPs

The AuNPs did not affect RAW cell viability across the concentration range assessed in the absence of LPS (Figure 2e). Conversely, in the presence of LPS, the AuNPs significantly (*p* < 0.001) decreased cell viability at concentrations ≥ 25 μg/mL. In the absence of LPS, compound **5** reduced (*p* < 0.001) cell viability to 40% pnly at the highest exposed concentration (100 μg/mL). In contrast, compound **5** in the presence of the mitogen LPS reduced (*p* < 0.001) cell viability by approximately 40% at concentrations ≥ 6.25 μg/mL.

#### 3.4.5. Isoliquiritin Apioside (**4**) and **4**@AuNPs

RAW cells exposed to the pure compound significantly (*p* = 0.003) reduced viability to 60 and 70% when exposed to 25 and 50 μg/mL in the presence of LPS (Figure 2g). The pure compound did not affect cell viability without LPS. AuNPs did not exert any detrimental effects on cell viability, regardless of the absence or presence of LPS.

#### 3.4.6. Liquiritin Apioside (**5**) and **5**@AuNPs

The AuNPs under basal conditions noticeably reduced (*p* < 0.001) cell viability in a dose-dependent manner at all concentrations assessed in the study (Figure 2i). Conversely, when stimulated with LPS, the nanoparticles exhibited a mitogenic effect, increasing (*p* < 0.001) cell viability at 25 μg/mL. They reduced (*p* < 0.001) viability only at 100 μg/mL. Without LPS, compound **5** significantly (*p* < 0.001) reduced viability by 40% at concentrations ≥ 50 μg/mL, with an approximate IC_50_ of 33.7 ± 2.5 μg/mL (Table 2). Exposure to compound **5** in the presence of LPS did not negatively impact cell viability, instead increasing (*p* = 0.005) cell viability at 25 μg/mL.

#### 3.4.7. Glabridin (**6**) and **6**@AuNPs

The AuNPs exhibited a noticeable (*p* < 0.001) decrease in cell viability at concentrations ≥ 50 μg/mL in both the unstimulated and LPS-stimulated conditions (Figure 2j), with approximate IC_50_ values of 38.2 ± 4.1 and 35.5 ± 2.1 μg/mL, respectively (Table 2). The percentage of viable cells was identified as 30% at concentrations of AUNPs ≥ 50 μg/mL. The unstimulated pure compound also produced a significant (*p* < 0.001) loss of viability at concentrations ≥ 50 μg/mL, with the percentage of viable cells ≤ 30% (IC_50_ ~ 28.4 ± 6.5 μg/mL). The LPS-stimulated compound **6** was cytotoxic (*p* < 0.001) at ≥25 μg/mL concentrations, with percentage viability ranging from 65 to 9%, respectively (IC_50_ ~ 27.2 ± 1.5 μg/mL).

#### 3.4.8. Total Extract (TE) and TE@AuNPs

When exposed to the cells, all the experimental concentrations of the extract and NP were not cytotoxic (0–100 μg/mL) (Figure 2l).

To summarize, the AuNPs conjugated with isoliquiritin apioside (**4**) were not toxic to the RAW cells across the concentration range in the presence or absence of LPS. Similarly, the TE alone was not toxic under all conditions and all the exposure concentrations. Comparably, the compounds liquiritin apioside (**5**) and isoliquiritin (**2**) did not hinder cell proliferation in the presence of LPS.

The RAW cells exposed to the pure compounds in the absence of LPS experienced different cytotoxic sensitivities: liquiritin (**1**) > liquiritin apioside (**5**) > glabridin (**6**) > neoisoliquiritin (**3**) > isoliquiritin (**2**). However, in the presence of LPS, the sensitivity of RAW cell viability differed from that of the unstimulated data. These sensitivities were neoisoliquiritin (**3**) > glabridin (**6**) > isoliquiritin apioside (**4**) > liquiritin (**1**).

The viability of the RAW cells was sensitive to three of the seven AuNPs in the absence of LPS: **5**@AuNPs > **2**@AuNPs > **6**@AuNPs. In the presence of LPS, the cells were sensitive to **3**@AuNPs > **2**@AuNPs > **6**@AuNPs > **1**@AuNPs > **5**@AuNPs.

### 3.5. Nitric Oxide Production

The NO assay based on the Griess reaction was used to assess the production of NO from the RAW cells after the exposure to the NP extract/pure compounds in either the absence or presence of LPS.

#### 3.5.1. Liquiritin (**1**) and **1**@AuNPs

Compound **1** and **1**@AuNPs did not elicit NO secretion from the RAW cells without LPS (Figure 2b). An anti-inflammatory response was seen when the cells were exposed to compound **1** in the presence of LPS. Compound **1** reduced (*p* < 0.001) the inflammatory activity at 25 μg/mL, with an approximate IC_50_ value of 16.5 ± 1.4 μg/mL (Table 4).

#### 3.5.2. Isoliquiritin (**2**) and **2**@AuNPs

RAW cells challenged with LPS, **2**@AuNPs reduced (*p* < 0.001) the inflammatory activity at 12.5 μg/mL (Figure 2d), with an IC_50_ ~ 18.2 ± 0.8 μg/mL (Table 4). Compound **2** and **2**@AuNPs under basal conditions did not affect the level of NO secreted by the cells.

#### 3.5.3. Neoisoliquiritin (**3**) and **3**@AuNPs

The **3**@AuNPs and compound in the presence or absence of LPS did not affect the level of NO secreted from the RAW cells (Figure 2f). 

#### 3.5.4. Isoliquiritin Apioside (**4**) and **4**@AuNPs

Isoliquiritin apioside under basal conditions did not elicit an inflammatory response from the RAW cells (Figure 2h). The AuNPs at basal levels induced (*p* < 0.001) NO production at 12.5 μg/mL. In the presence of LPS, compound **4** and **4**@AuNP did not impact NO secretion from the RAW cells. 

#### 3.5.5. Liquiritin Apioside (**5**) and **5**@AuNPs

The level of NO secreted from the cells was not evaluated as **5**@AuNP was shown to be cytotoxic (Figure 2i).

#### 3.5.6. Glabridin (**6**) and **6**@AuNPs

The AuNPs and the pure compound did not affect NO production without LPS (Figure 2k). However, in the presence of LPS, both the AuNPs and compound **6** reduced (*p* < 0.001) the production of NO from the RAW cells at concentrations ≥ 12.5 μg/mL, indicating anti-inflammatory activity. Compound **6** exhibited an approximate IC_50_ value of 27.5 ± 1.5 μg/mL in the presence of LPS (Table 4). 

#### 3.5.7. Total Extract (TE) and TE@AuNPs

The TE-capped AuNPs induced an inflammatory response (*p* < 0.001) under basal conditions at concentrations ≥ 50 μg/mL (Figure 2m). This increase in NO production was seven times more than that of the negative control (0 μg/mL -LPS). TE@AuNPs did not impact NO production in the presence of LPS. The production of NO was not affected by the TE in either the absence or presence of LPS.

Without LPS, neither the TE nor any of the pure compounds affected NO secretion. In the absence of LPS, NO secretion was modulated by the TE@AuNPs and **4**@AuNPs. In the presence of LPS, the TE did not affect NO secretion. However, the pure compounds liquiritin (**1**) and glabridin (**6**) inhibited NO secretion in the presence of LPS. The NO secretion in the presence of LPS was inhibited by all the compounds conjugated to AuNPs, except liquiritin (**1**), neoisoliquiritin (**3**), isoliquiritin apioside (**4**), and the TE. 

The anti-inflammatory potential of the compounds ranged within the order liquiritin (**1**) ≤ glabridin (**6**) in the presence of LPS. The anti-inflammatory potential of the compounds conjugated to the AuNPs ranged within the order **4**@AuNPs ≤ TE@AuNPs in the absence of LPS, and **1**@AuNPs ≤ **6**@AuNPs ≤ **2**@AuNPs in the presence of LPS.

## 4. Discussion

The synthesis of metal NPs using biological materials, mainly plant extracts, is widely accepted as an eco-friendly method to avoid more contamination of the environment. AuNPs show excellent safety margins and low toxicity in vitro and in vivo, allowing a wide range of applications in the biomedical field.

Plant extracts are a complex mixture of chemical molecules of different natures and functionalities. In the context of metallic NP preparation, total plant extracts are less preferable due to the complex nature of the multicomponent capping surrounding the metal core. Employing a single-molecule approach, particularly in medical applications, is highly recommended as it offers superior advantages.

Natural compounds have a positive impact on human health. When utilized as capping agents, these natural products not only reduce the metal precursors into their metallic form but also enhance the stability and longevity of the resulting metal NPs. Furthermore, employing natural products as capping agents confers additional advantages, such as improved targeting capabilities of AuNPs towards specific tissues or organs, thus enhancing their smartness. During the process of nanoparticle formation, only a small portion of the active compound(s) undergoes conjugation in the nanoform. Despite this conjugated form having a lower concentration, it has been reported to exhibit enhanced activity compared with the intact extract and active compound(s). Additionally, AuNP conjugates serve as excellent compositions to enhance the bioavailability of natural compounds while mitigating their potential toxicity [48,49].

In this work, six major compounds from licorice were isolated and used as reducing, capping, and stabilizing agents for AuNPs. The SPR bands of the prepared AuNPs ranged between 538–556 nm (Figure S3 and Table 2). The values are within the acceptable published results for AuNPs; for example, mangiferin- and hypoxoside-capped AuNPs’ SPR bands were 527 and 534 nm, respectively [17,50]. 

The ZP of the particles ranged from −9.1 (for **4**@AuNPs) to −32.7 mV (**6**@AuNPs), as indicated (Table 2); compound **6** conjugated AuNPs were the most stable, followed by **3**@AuNPs (−32.3), then **1**@AuNPs (−29.9). The observation of the low stability of **5**@AuNPs (−17.1 mV) compared with **1**@AuNPs (-29.9 mV) is interesting since compound **5** is more hydrophilic with one sugar unit extra; the same behavior was also observed between **2**@AuNPs (−20.2) and **4**@AuNPs (−9.1). Currently, we lack a precise explanation for this behavior, apart from attributing it to the steric effect caused by the presence of bulky sugar units in the case of **4**@AuNPs and **5**@AuNPs. It is plausible that these bulky groups impede interactions on the metal surface and/or hinder the charge transfer to the capping layers around the core of the metal nanoparticles (NPs). This proposal partially supports the stability observed in the case of **6**@AuNPs, which features **6** as the aglycone, devoid of such bulky sugar units [45]. On the other hand, the hydrodynamic size supports the proposed explanation, where the size of **1**@AuNPs (184.0 nm) > **5**@AuNPs (79.31 nm) and **2**@AuNPs (221.9 nm) > **4**@AuNPs (184.0 nm).

The TEM analysis (Figure S5) showed comparable small sizes for all samples. These results indicate the fast electron donation of the compounds during the reduction process. TEM analysis also showed polymorphism of all AuNPs formed, and different shapes appeared in the figures. The size variation of the AuNPs can be attributed to the different interaction modes of the phenolics during the reducing and stabilization steps. Polyhydroxylated natural compounds, especially polyphenols, are well known for this behavior under neutral conditions; for example, AuNPs conjugated with mangiferin [14], quercetin [15], proanthocyanidin dimer [16], hypoxoside [17,18], and hesperidin [19,20] showed polymorphic nature.

The XRD pattern (Figure S8) indicated that the biosynthesized gold nanoparticles were crystalline. The average particle size was calculated from the XRD peaks by applying the Scherrer equation. The relative sizes had a range of 5.48–10.06 nm. 

**Figure 3:**
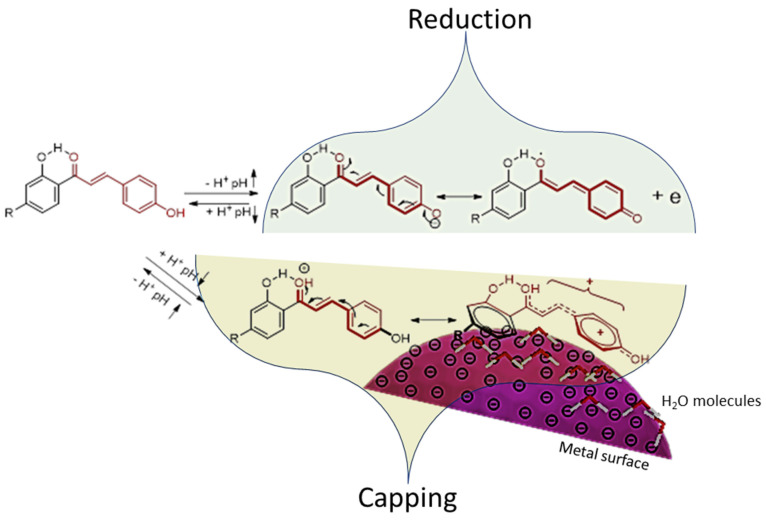
A proposed mechanism for the reduction and capping of chalcones isolated in this study (represented by compound **3**). The reduction process includes the loss of an electron and this step is pH-dependent. The second step is the capping process where the compound forming a shell may be two or three layers around the metal NPs.

It is important to note that the sizes of AuNPs nanoparticles obtained via different methods varied. The DLS method produced a size range of 79 to 350 nm, while HRTEM yielded a range of 16 to 83 nm (Figure S5) and XRD indicated a range of 5 to 10 nm (see Table 2). DLS measurements consider the hydrodynamic size, including the capping agent shells around the metal core, which can introduce errors in determining the actual average particle size. These errors can be up to five times larger than those from TEM. HRTEM measures the actual size related to the metal core of the nanoparticle, but it has the limitation of not necessarily representing the entire population of nanoparticles since the selected image may not be representative. The most accurate method for determining relative particle sizes is through XRD. On the other hand, DLS demonstrates the ability of the metal core to attract more molecules from the surrounding medium, which could contribute to the payload, bioactivity, and stability of the nanoparticles. Figure 3 shows the possible mechanism of formation of NPs, using chalcone pharmacophore as an example. 

Figures S9 and S10 show the typical FTIR spectra of each compound with the corresponding AuNPs. Some synthesized AuNPs showed similarities with their respective compounds, confirming the capping agent’s presence around the metal NP core [51]. 

Significantly, it should be noted that the FTIR spectra of compounds **2** and **4** (and their NPs) exhibit similarity due to their shared pharmacophore. The only difference exists in the glycosidic side chain, which has a minimal impact on the characteristics of both compounds. An analogous scenario also applies to compounds **1** and **5**. 

The stability study of the formed AuNPs is essential to understanding the expected potency and the shelf life of the synthesized AuNPs in different biogenic media. Most of the NPs, notably **4**@AuNPs, **5**@AuNPs, **6**@AuNPs, and TE@AuNPs, demonstrated stable color across various media, and the SPR remained the same in all the samples. However, certain NPs, namely **1**@AuNPs, **2**@AuNPs, and **3**@AuNPs, exhibited lower stability in cysteine. Additionally, the last one (**3**@AuNPs) was found to be influenced by glycine. After 24 h of incubation with BSA, most of the NPs exhibited an increase in their absorbance bands. This observed increment can be attributed to the direct interaction between the phenolic hydroxyl groups of the AuNP conjugates and the amino acids. Furthermore, BSA contains thiol groups that can interact with the metal surface, providing an additional factor contributing to the observed enhancement. AuNPs synthesized using procyanidin dimer showed the same effect [52]. 

Due to its diverse pharmacological effects, licorice has been used medicinally in South-East Asia/East Asia/the Middle East/Asia [53,54]. The plant has several physiologically active compounds, with the following being the most abundant: triterpenoids, flavonoids, and polysaccharides [55]. These compounds are believed to be responsible for pharmacological effects, including antitumor, antiviral, anti-inflammatory, cardiovascular protection, antitussive, hepatoprotection, immunoregulatory, neuroprotective, and skin-protective effects [56,57,58]. It demonstrates notable effects, including its role as an antidepressant [51,52,53,54], neuroprotective agent [49,52,55,56], anticancer compounds [57,58], and anti-inflammatory active agent.

Liquiritin, a compound extracted from licorice, is known for its diverse range of pharmacological activities. It demonstrates antidepressant [59,60,61,62], neuroprotective [57,60,63,64], anticancer [65,66], and anti-inflammatory activities [2,55,61]. Additionally, isoliquiritin has been found to have antidepressant [62], antiangiogenic [67], neuroprotective [68], and wound healing [69] properties. Neoisoliquiritin has been identified for its antitumor effects [70,71], and isoliquiritin apioside has demonstrated antigenotoxic [71] and antiangiogenic [72] activities. Liquiritin apioside has proven effective in protecting against epithelial injury in chronic obstructive pulmonary disease [73], and has antitussive [74] properties. Lastly, glabridin has shown anti-inflammatory, anti-atherogenic, estrogenic-like effects and a high capacity to regulate energy metabolism [75]. 

Macrophages are among the first immune cells that pathogens encounter upon entering the body. As a result, the body experiences an inflammatory response, leading to the re-establishment of normal tissue structure and function [76]. Therefore, the RAW 264.7 cell line was selected to evaluate the bioactivity of the licorice extract, compounds, and nanoparticles, as the cell line has been used to predict the potential effect of natural products for their bioactivity on primary cells or in vivo [77].

This study, to our knowledge, was the first time the total extract, isolated compounds, and their respective green synthesized AuNPs, liquiritin (**1**), isoliquiritin (**2**), neoisoliquiritin (**3**), isoliquiritin apioside (**4**), liquiritin apioside (**5**), and glabridin (**6**) were all screened in one study to assess their potential effects on cell viability and the inflammatory biomarker, NO, using murine cell line RAW 264.7 macrophages.

The TE was not cytotoxic nor did it exhibit any effect on inflammation at all concentrations under both conditions (Figure 2). Comparably, under basal conditions, the AuNPs increased NO secretion from the cells at concentrations ≥ 50 μg/mL (Figure 2m). It is considered that AuNPs induces inflammation in RAW cells by mediating reactive oxygen species (ROS) and nuclear factor κβ (NF-κβ) signaling pathways which lead to the production of inflammatory cytokines such as COX-2, IL-6, and TNF-α [78]. The induction of NO under basal conditions was also seen at **4**@AuNP (≥12.5 μg/mL). Although the total extract was non-toxic and did not affect inflammation, the isolated compounds and their respective AuNPs showed various degrees of cytotoxicity and inflammatory responses. The nontoxic effects of the total extracts explain the wide range of activity, especially antiviral and anti-inflammatory, as mentioned above. From the chemical point of view, the presence of a plethora of pharmacologically active compounds in the extract makes it behave differently from the isolated pure compounds described in this study; other compounds such as triterpenoids and flavonoids may play a role in determining the ultimate biological activity.

The compounds liquiritin (**1**) and liquiritin apioside (**5**) decreased cell viability at concentrations ≥ 50 μg/mL in the absence of LPS (Figure 2a,i). A similar trend was seen when liquiritin (**1**) was exposed to human liver cells, HepG2, for 24 hrs. The authors demonstrated that HepG2 viability was decreased to below 40% at concentrations ≥ 120 μM. Chen et al. [79] also reported a decrease in cell viability when PC12 cells in the presence of neuronal growth factor were exposed to liquiritin over 3- and 5-day periods at concentrations ≥ 50 μg/mL [63]. Liquiritin apioside (**5**) did not affect RAW cell proliferation under a simulated inflammatory response (Figure 2i). However, cell proliferation was hampered in the absence of LPS at concentrations ≥ 50 μg/mL. Although not assessed in the study, it is proposed that liquiritin (**1**), and possibly liquiritin apioside (**5**), induces cell apoptosis by activating the caspases, setting off a cascade reaction that subsequently coordinates cell death as seen in cell lines such as A549 and rheumatoid arthritis fibroblast-like synoviocytes [56,61,65,80]. 

When **1**@AuNPs were exposed to the RAW cells, the level of cytotoxicity decreased, as it was exhibited only at 100 μg/mL in the presence of LPS (Figure 2a), depicting the mitogenic effect of LPS. Studies by Weng et al. and Wang et al. established a liquiritin-loaded micelle and liquiritin-loaded precursor liposome, where the bioavailability was greater than liquiritin itself, in mice and rat models, respectively, which could explain the above phenomena, albeit in vitro [81,82]. Conversely, when **5**@AuNPs were exposed to RAW cells, they exhibited more significant toxicity than **1**@AuNPs as they were toxic at concentrations ≥ 3.125 μg/mL and 100 μg/mL in the absence or presence of LPS. This could be due to the enhanced uptake of the particles into the cell. This observation may support the direct effect of AuNPs for enhancing the activity of natural compounds even if the compound is hydrophilic. 

Isoliquiritin (**2**) alone reduced cell viability at 100 μg/mL in the absence of LPS, with no effect on viability when stimulated with LPS (Figure 2c). Zhou and Ho found that isoliquiritin upregulated Bax and Bid proteins as well as increasing caspase activity in A549 lung fibroblasts [80]. This could be attributed to what was seen at 100 μg/mL. It was also found that exposure of isoliquiritin to B65 neuroblastomas reduced viability at 100 μM after 48 h [57], whereas two studies reported that RAW 264.7 cell viability was not affected when exposed to 0–1.6 μM or 0–200 μM isoliquiritin (**2**) for 24 h, contradicting the findings in this study [76,83]. This discrepancy in viability could be attributed to the differences in concentration ranges assessed in our study. Isoliquiritin (**2**) possesses a sugar moiety, thereby reducing its lipophilicity and compatibility with the RAW cellular membrane, as the affinity for the cell membrane plays a role in the uptake of lipophilic compounds by passive diffusion [76]. However, when isoliquiritin (**2**) was conjugated to the AuNPs, the level of cytotoxicity increased at concentrations ≥ 12.5 μg/mL and ≥50 μg/mL when unstimulated or stimulated by LPS, respectively (Figure 2c). The observed activity of the conjugated **2**@AuNPs supports the role of AuNPs in increasing the activity of the capping agent, most probably through the formation of a proactive form at the surface of the magnetic nanoparticles (MNPs), thereby making it more compatible with the cell membrane and promoting the uptake of the NPs.

The chalcone glycoside, isoliquiritin apioside (**4**), did not impact cell viability in the absence of LPS. These results were corroborated by Kim and Ma, who found that up to 100 μM of isoliquiritin apioside (**4**) did not affect cell proliferation of human epithelial cells (HT1080) [72]. However, when in the presence of LPS, viability was reduced between 60 and 70% when exposed to 25 and 50 μg/mL isoliquiritin apioside (Figure 2g). An extensive review by Dhaliwal et al. stipulated that chalcones such as isoliquiritin apioside (**4**) can cause loss of cell viability and mitochondrial membrane potential while inducing morphological changes consistent with apoptosis [84]. Further tests are needed to explain the reduced viability at the concentrations mentioned above. The non-toxic nature of isoliquiritin apioside (**4**) was further compounded when attached to the AuNPs, as proliferation was not impacted across all concentrations and under all conditions assessed.

The prenylated isoflavonoid glabridin (**6**) is a phytoestrogen and acts via estrogen receptors, which RAW 264.7 cells have been shown to express [75,85]. This would indicate the route via which the compound is taken into the cells and exerts its toxicity. This was seen at concentrations > 25 μg/mL of glabridin (**6**) and glabridin conjugated to the AuNPs (Figure 2j). Cellular experiments have shown that glabridin induces cancer cell apoptosis by activating the mitochondrial apoptotic pathway and the caspase cascade [86]. However, evidence has been contradictory as glabridin promoted MC3T3-E1 cell proliferation and neuroprotection [87].

In brief, the RAW cells exposed to the purified components of licorice without LPS experienced different cytotoxic sensitivities: liquiritin (**1**) > liquiritin apioside (**5**) > glabridin (**6**) > isoliquiritin (**2**). However, in the presence of LPS, the sensitivity of RAW cell viability differed from that of the unstimulated samples. These sensitivities were glabridin (**6**) > isoliquiritin apioside (**4**) > liquiritin (**1**). The RAW cells were sensitive to three of the seven AuNPs in the absence of LPS: **5**@AuNPs > **2**@AuNPs > **6**@AuNPs. In the presence of LPS, the cells were sensitive to **2**@AuNPs > **6**@AuNPs > **1**@AuNPs > **5**@AuNPs.

When the RAW cells were challenged with LPS and subsequently treated with the various compounds, the inflammatory marker NO was monitored at non-toxic concentrations. The reduction of NO by the compounds occurred at different concentrations: liquiritin (≥25 μg/mL) and glabridin (≥12.5 μg/mL) (Figure 2). Interestingly, the methanolic extract did not reduce inflammation in comparison to the isolated compounds. These results are in line with the reported anti-inflammatory properties of the compounds. Liquiritin was shown to reduce the phosphorylation of NF-κβ when THP-1 monocytic cells were stimulated with LPS. This subsequently reduced the monitored inflammatory markers IL-6, TNF-, and IL-1 [58]. Gao et al. reported that liquiritin inhibited protein and mRNA levels of the inflammatory cytokines IL-6 and IL-8 in IL-1 in stimulated SW982 human synovial cells [55]. They showed that liquiritin can suppress inflammation across cell types, and Wang et al. further compounded this as NF-κβ was reduced in HepG2 cells [79]. Chen et al. provided insight into the mechanism employed by liquiritin apioside in RAW 264.7 cells as they were shown to suppress the PI3K/AKt/NF-κβ pathways responsible for inflammation [70]. By inhibiting this pathway in macrophages, the expression of inducible nitric oxide synthase (iNOS) and NO production in LPS-induced RAW 264.7 cells was inhibited [86]. Subsequent studies have also reported the decrease of inflammation via inhibiting the NF-κβ pathway by the other compounds: isoliquiritin, isoliquiritin apioside, and glabridin [72,75,83,84] 

The subsequent AuNPs conjugated to the various compounds also reduced inflammatory activity: **2**@AuNPs (12.5 μg/mL) and **6**@AuNPs (≥12.5 μg/mL) (Figure 2). The reduction in inflammation associated with the NPs is postulated to occur via the NF-κβ pathway, as previously mentioned in the discussion. This can be inferred through other limited studies monitoring the anti-inflammatory activity on RAW cells exposed to other green synthesized AuNPs. These studies noted that Euphrasia officinalis, Suaeda japonica and chitosan–ginsenoside compound K (CK) AuNPs reduced inflammatory activity by the following mechanisms: inhibiting the iNOS and COX-2 mRNA levels; decreasing the phosphorylation and degradation of inhibitor kappa beta; decreasing the nuclear translocation of NF-κβ 65 and p50, and inhibiting the JAK/STAT pathway [88,89]. In certain cases, the NPs reduced inflammation at lower concentrations, as in the case of **2**@AuNPs. This could be due to the increased uptake of the AuNPs by the RAW cells. Further studies will be needed to explore and establish how the NPs are taken up and, if so, where they are located in the cell. 

In synopsis, when challenged with LPS, the anti-inflammatory potential of the compounds ranged in the order liquiritin (**1**) ≤ glabridin (**6**). The anti-inflammatory potential of the compounds conjugated to the AuNPs ranged in the order **6**@AuNPs ≤ **2**@AuNPs in the presence of LPS.

There were limitations to this study as it was a pilot study. In future studies, we aim to monitor cellular uptake and the intracellular morphology of cells exposed to AuNPs. In addition, other inflammatory biomarkers and oxidative stress biomarkers will be assessed.

## 5. Conclusions

Phenolic compounds can form, stabilize, and activate AuNPs due to their flexibility in donating electrons and the formation of proactive forms at the surface of the AuNPs. The interaction at the surface of the nanoparticles caused a shift in activity for some of the AuNP conjugates from their intact capping agents, as shown by liquiritin in this study. In most cases, the individual compounds exhibited a more potent anti-inflammatory response than the total methanolic extract or their AuNP counterpart. Although certain compounds exhibited a greater anti-inflammatory response, they were also more cytotoxic than the extract and their relative AuNPs. As suggested by the results, the 2@AuNP conjugate exhibited the highest anti-inflammatory potency compared with the other samples, necessitating further investigation to elucidate its potential as a candidate for inflammation treatment. Utilizing individual, well-characterized natural products with established pharmacological properties represents a significant alternative to employing entire extracts. This approach allows precise control over variable factors and facilitates the design and prediction of metallic nanoparticles.

## Figures and Tables

**Table 1:** ^1^H and ^13^C NMR spectroscopic data for compounds **1**–**6** *.

	Liquiritin (1)		Liquiritin Apioside (5)		Glabridin (6)			Isoliquiritin (2)		Neoisoliquiritin (3)		Isoliquiritin Apioside (4)	
	d_C_	d_H_ (*multi*, *J*)	d_C_	d_H_ (*multi*, *J*)	d_C_	d_H_ (*multi*, *J*)		d_C_	d_H_ (*multi*, *J*)	d_C_	d_H_ (*multi*, *J*)	d_C_	d_H_ (*multi*, *J*)
**1**	-	-	-	-		-	**C=O**	191.7	-	192.5	-	191.1	-
**2**	78.6	5.52 (*br d*,13.0)	78.9	5.51 (*d*, 2.0, 13.0)	70.02	4.20 (*d*, 10.0) 3.85 (*d*, 10.0)	**α**	119.6	7.87 (*d*, 15.5)	117.7	7.82 (*d*, 16.6)	119.2	7.86 (*d*, 15.8)
**3**	43.2	3.12 (*dd*, 16.6, 13.0) 2.66 (*br d*, 16.6)	43.1	3.11 (*dd*, 16.0, 13.0) 2.26 (*br d*, 16.0)	31.60	3.33 *m*	**β**	143.8	7.77 (*d*, 15.5)	145.5	7.79 (*d*, 16.6)	143.2	7.76 (*d*, 15.8)
**4**	189.9	-	189.9	-	30.36	2.64 (*d*, 15.3) 2.84 (*dd*, 3.1, 15.3)	**1**	133.4	-	125.9	-	128.4	-
**5**	128.4	7.63 (*d*, 8.6)	128.1	7.64 (*d*, 9.0)	127.8	6.15 (*d*, 8.2)	**2**	128.8	7.86 (*d*, 8.8)	132.9	7.79 (*d*, 8.4)	130.7	7.86 (*d*, 8.8)
**6**	110.8	6.36 (*d*, 2.0)	110.8	6.51 (*dd*, 9.0, 1.2)	109.5	6.69 (*d*, 8.2)	**3**	116.9	7.10 (*d*, 8.8)	116.4	6.85 (*d*, 8.4)	116.3	7.07 (*d*, 8.8)
**7**	165.2	-	165.1	-	151.8	-	**4**	159.8	-	161.2	-	159.2	-
**8**	102.6	6.51 (*d*, 2.0, 8.6)	102.6	6.35 (*d*, 1.2)	106.8	-	**5**	116.9	7.10 (*d*, 8.8)	116.4	6.85 (*d*, 8.4)	116.3	7.07 (*d*, 8.8)
**9**	163.1	-	163.1	-	149.7	-	**6**	128.8	7.86 (*d*, 8.8)	131.9	7.78 (*d*, 8.4)	130.7	7.86 (*d*, 8.8)
**10**	113.3	-	113.3	-	114.7	-	**1′**	113.2	-	115.2	-	112.6	-
**1′**	132.4	-	132.4	-	118.3	-	**2′**	166.3	-	163.9	-	165.9	-
**2′**	128.0	7.44 (*d*, 8.6)	128.4	7.44 (*d*, 8.5)	155.9	-	**3′**	103.0	6.25 (*d*, 1.7)	103.9	6.58 (*d*, 1.8)	102.6	6.28 (*d*, 2.0)
**3′**	116.2	7.06 (*d*, 8.5)	116.0	7.04 (*d*, 8.5)	102.6	6.33 (*d*, 2.3)	**4′**	166.3	-	165.6	-	165.9	-
**4′**	157.4	-	157.3	-	157.1	-	**5′**	108.9	6.41 (*dd*, 1.7, 9.0)	108.6	6.62 (*dd*, 1.8, 9.0)	108.6	6.25 (*d*, 2.0)
**5′**	116.2	7.06 (*d*, 8.5)	116.0	7.04 (*d*, 8.5)	108.3	6.14 (*dd*, 2.3, 8.4)	**6′**	131.2	8.18 (*d*, 9.0)	131.9	8.27(*d*, 9.0)	132.9	8.17 (*d*, 9.0)
**6′**	128.0	7.44 (*d*, 8.6)	128.1	7.44 (*d*, 8.5)	128.7	6.81 (*d*, 8.4)	**1″**	100.3	4.98 (*d*, 8.8)	100.0	5.04 (*d*, 8.6)	98.3	5.05 (*d*, 7.4)
**1″**	100.3	4.89 (*d*, 7.3)	98.6	4.96 (*d*, 7.2)	116.8	6.48 (*d*, 9.8)	**2″**	73.6	3.29 (*d*, 8.6)	73.5	3.24 (*d*, 7.4)	79.3	3.74 (*br s*)
**2″**	73.2	3.26 ^a^ (*m*)	75.7	3.74 (*s*)	129.2	5.47 (*d*, 9.8)	**3″**	77.0	3.30 (*t*, 8.6 )	76.9	3.31 (*t*, 9.0)	76.9	3.49 ^a^
**3″**	77.0	3.33 ^a^	77.1	3.36 ^a^	75.1		**4″**	70.1	3.17 (*t*, 8.8)	70.0	3.17 (*t*, 8.4 )	69.9	3.19 (*t*, 7.5)
**4″**	69.7	3.17 (*dd*, 8.9, 10.0)	69.9	3.95 (*d*, 9.4)	26.6	1.23 (*s*)	**5″**	77.6	3.34 (*dd*, 9.4, 7.6)	77.6	3.34 (*dd*, 9.0, 7.0)	77.1	3.42 ^a^
**5″**	76.6	3.28 ^a^	76.9	3.49 ^a^	26.6	1.23 (*s*)	**6″**	61.1	3.69 (*d*, 11.0) 3.18 (*dd*, 8.0, 11.0)	61.0	3.69 (*d*, 11.0) 3.34 (*dd*, 11.0, 7.0 )	60.5	3.45 ^a^ 3.70 (*br d*, 10.9)
**6″**	60.7	3.69 (*d*, 11.4) 3.47 (*dd*, 5.5, 11.4)	60.6	3.45 ^a^ 3.69 (*br d*, 11.6)			**1** **‴**					108.6	5.36 (*br s*)
**1** **‴**			108.7	5.36 (*s*)			**2** **‴**					76.0	3.51 (*d*, 7.4)
**2** **‴**			77.6	3.48 ^a^			**3** **‴**					79.3	-
**3** **‴**			79.3	-			**4** **‴**					74.0	3.65 (*d*, 9.5) 3.94 (*d*, 9.5)
**4** **‴**			74.0	3.65 (*d*, 9.3) 3.94 (*d*, 9.3)			**5** **‴**					64.3	3.31 (*s*, 2H)
**5** **‴**			64.3	3.33 (*s*, 2H)									

* Spectra were measured in DMSO- *d*_6_ except compound (**6**) in acetone. NMR operated at 400 MHz (^1^H) and 100 MHz (^13^C), *multi*: multiplicity, *J* values in *Hz*, *s* singlet, *br s* broad singlet, *d* doublet, *dd* doublet of doublet, *br d* broad doublet, *t* triplet, *m* multiple; ^a^ overlapped signals in the same column.

**Table 2:** Particle sizes, zeta potential, polydispersity index, UV absorbance, and HRTEM average size measurements of different synthesized AuNPs.

Sample	Zeta Potential (mV)	Pdi	Hydrodynamic Size (nm)	UV–Vis λ Max (nm)	Average Size from XRD * (nm)
**1**@AuNPs	−29.9	0.12	184.0	551	6.56
**2**@AuNPs	−20.2	0.26	221.9	547	5.48
**3**@AuNPs	−32.3	0.12	351.0	550	10.02
**4**@AuNPs	−9.10	0.22	184.0	553	9.24
**5**@AuNPs	−17.1	0.34	79.31	556	10.06
**6**@AuNPs	−32.7	0.19	257.8	544	10.02
TE@AuNPs	−27.7	0.23	320.0	538	10.06

* Values obtained using the Scherrer equation (see Table S1).

**Table 3:** Approximate cell viability IC_50_ values (µg/mL) of the respective compounds and their AuNPs conjugates ± SEM.

	−LPS		+LPS	
Sample	Comp.	AuNP	Comp.	AuNP
Comp. **1**	39.7 ± 1.6	-	60.5 ± 4.5	-
Comp. **2**	-	29.2 ± 2.3	-	-
Comp. **3**	-	-	-	-
Comp. **4**	-	-	-	-
Comp. **5**	47.8 ± 0.2	33.7 ± 2.5	-	-
Comp. **6**	28.4 ± 6.5	38.2 ± 4.1	27.2 ± 1.5	35.5 ± 2.1
TE	-	-	-	-

**Table 4:** Approximate NO IC_50_ values (µg/mL) of the respective compounds and their AuNPs conjugates ± SEM.

	−LPS		+LPS	
Sample	Comp.	AuNP	Comp.	AuNPs
Comp. **1**	-	-	16.5 ± 1.4	-
Comp. **2**	-	-	-	18.2 ± 0.8
Comp. **3**	-	-	-	-
Comp. **4**	-	-	-	-
Comp. **5**	-	-	-	-
Comp. **6**	-	-	27.5 ± 1.5	-
TE	-	-	-	-

## Data Availability

The 2D NMR spectra will be made available by the corresponding author on request.

## Supplementary Materials

The following supporting information can be downloaded at https://www.mdpi.com/article/10.3390/jfb15040095/s1. Figure S1. LC-MS chromatogram of the total extract and the tentative identification of the major compounds [90,91,92,93,94,95]. Figure S2.1. ^1^H and ^13^C spectra of compound **1**. Figure S2.2. ^1^H and ^13^C spectra of compound **2**. Figure S2.3. ^1^H and ^13^C spectra of compound **3**. Figure S2.4. ^1^H and ^13^C spectra of compound **4**. Figure S2.5. ^1^H and ^13^C spectra of compound **5**. Figure S2.6. ^1^H and 13C spectra of compound **6**. Figure S3. Ultraviolet-visible spectra of the green synthesized AuNP conjugates and the intact extract/pure compounds. Figure S4. Zeta potential and relative size distribution of the synthesized NPs. Figure S5. HRTEM of the synthesized AuNPs. Figure S6. Stability of the AuNP conjugates in different biogenic media after 24 h. Figure S7. Stability of the AuNP conjugates for three months. Figure S8. XRD of the synthesized AuNPs. Figure S9. FTIR spectra of the synthesized AuNPs with their intact compounds. Figure S10. FTIR of total extract/pure compounds (black) and their corresponding AuNPs (red) in the 1700–930 cm^−1^ range. Table S1. The calculation using Scherrer equation.
