# Supplementary material for: Green Synthesis of Gold Nanoparticles Using Liquiritin and Other Phenolics from Glycyrrhiza glabra and Their Anti-Inflammatory Activity
Source: J Funct Biomater. 2024 Apr 6;15(4):95. doi: 10.3390/jfb15040095 (PMC11051159; doi:10.3390/jfb15040095)
Supplement: Supplementary file 1 [file jfb-15-00095-s001.zip › jfb-2915912-supplementary_A.pdf]

Journal of *Functional Biomaterials*

Supplementary Materials

Green Synthesis of Gold Nanoparticles Using Liquiritin and Other Phenolics from Glycyrrhiza Glabra and Their Anti-Inflammatory Activity

Ali O. E. Eltahir [1], Kim L. Lategan [2], Oladipupo M. David [2], Edmund J. Pool [2], Robert C. Luckay [3] and Ahmed A. Hussein [1,*]

1 Chemistry Department, Cape Peninsula University of Technology, Bellville 7535, South Africa; aliomers250@gmail.com
2 Department of Medical Bioscience, University of Western the Cape, Bellville 7535, South Africa; klategan@uwc.ac.za (K.L.L.); 3681075@myuwc.ac.za (O.M.D.); epool@uwc.ac.za (E.J.P.)
3 Department of Chemistry and Polymer Science, Stellenbosch University, Stellenbosch 7602, South Africa; rcluckay@sun.ac.za
* Correspondence: mohammedam@cput.ac.za

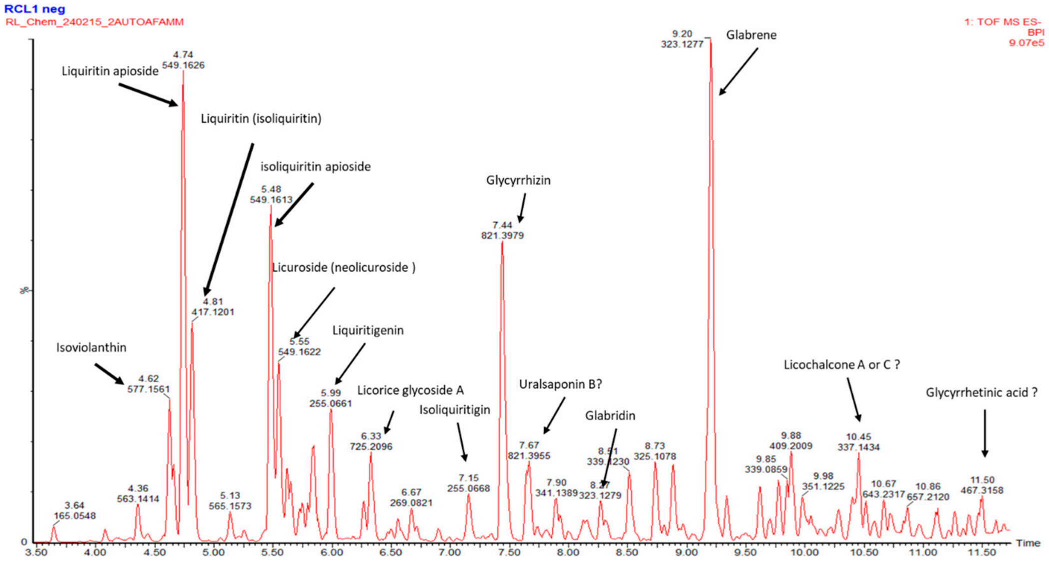

Figure S1. LC-MS chromatogram of the total extract and the tentative identification of the major compounds [90–95].

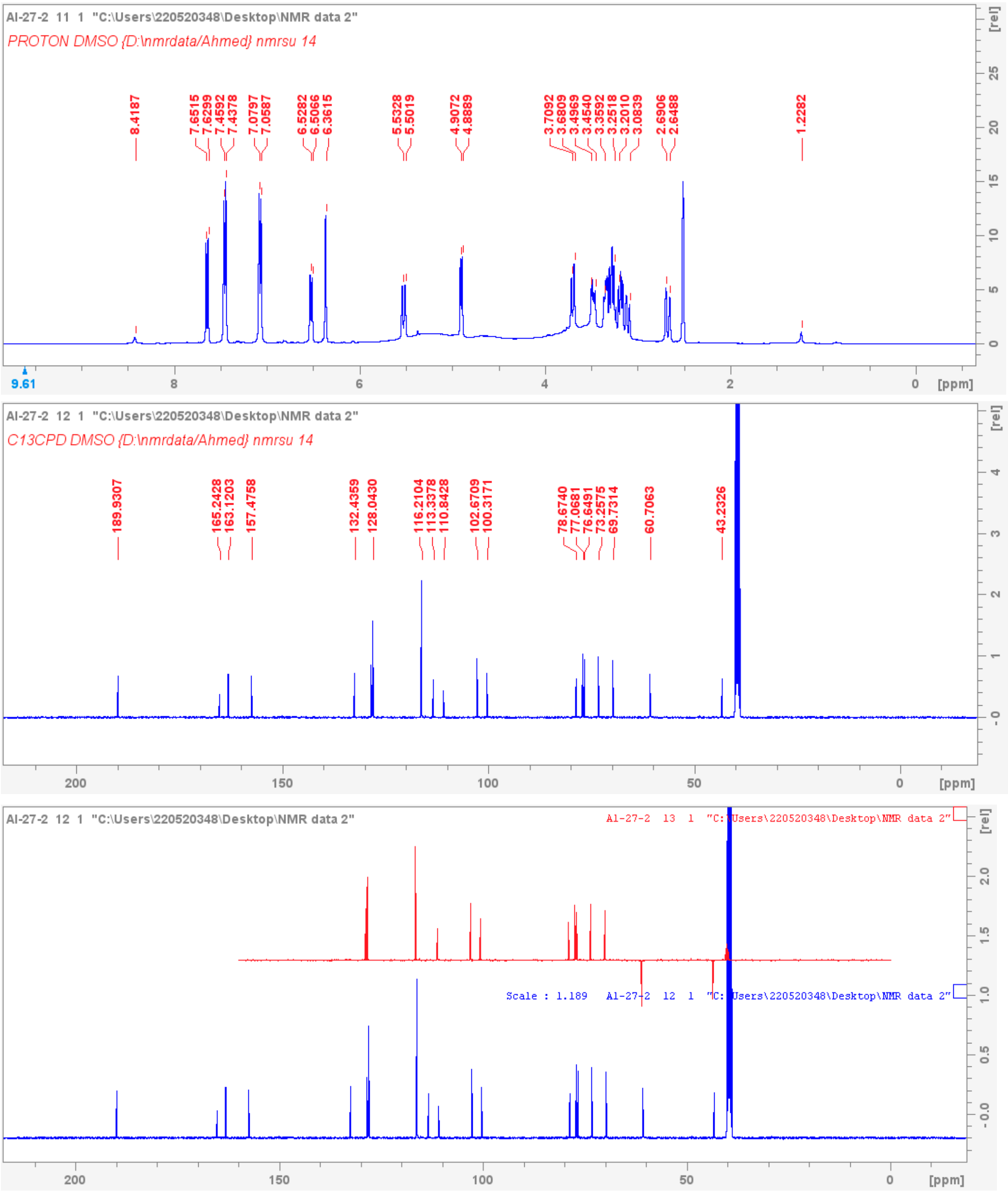

Figure S2.1. ^{1}H and ^{13}C spectra of compound **1**.

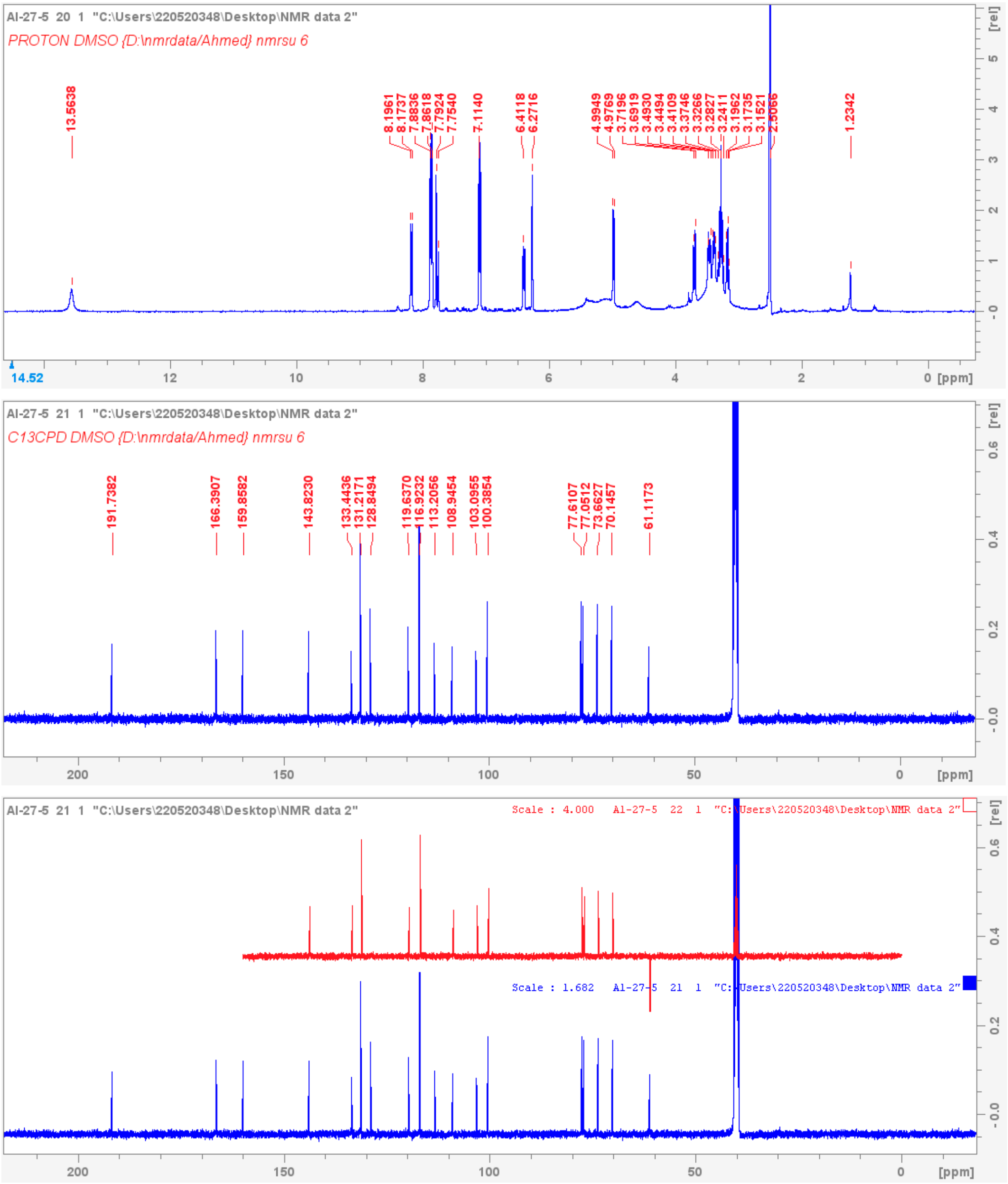

Figure S2.2. ^{1}H and ^{13}C spectra of compound **2**.

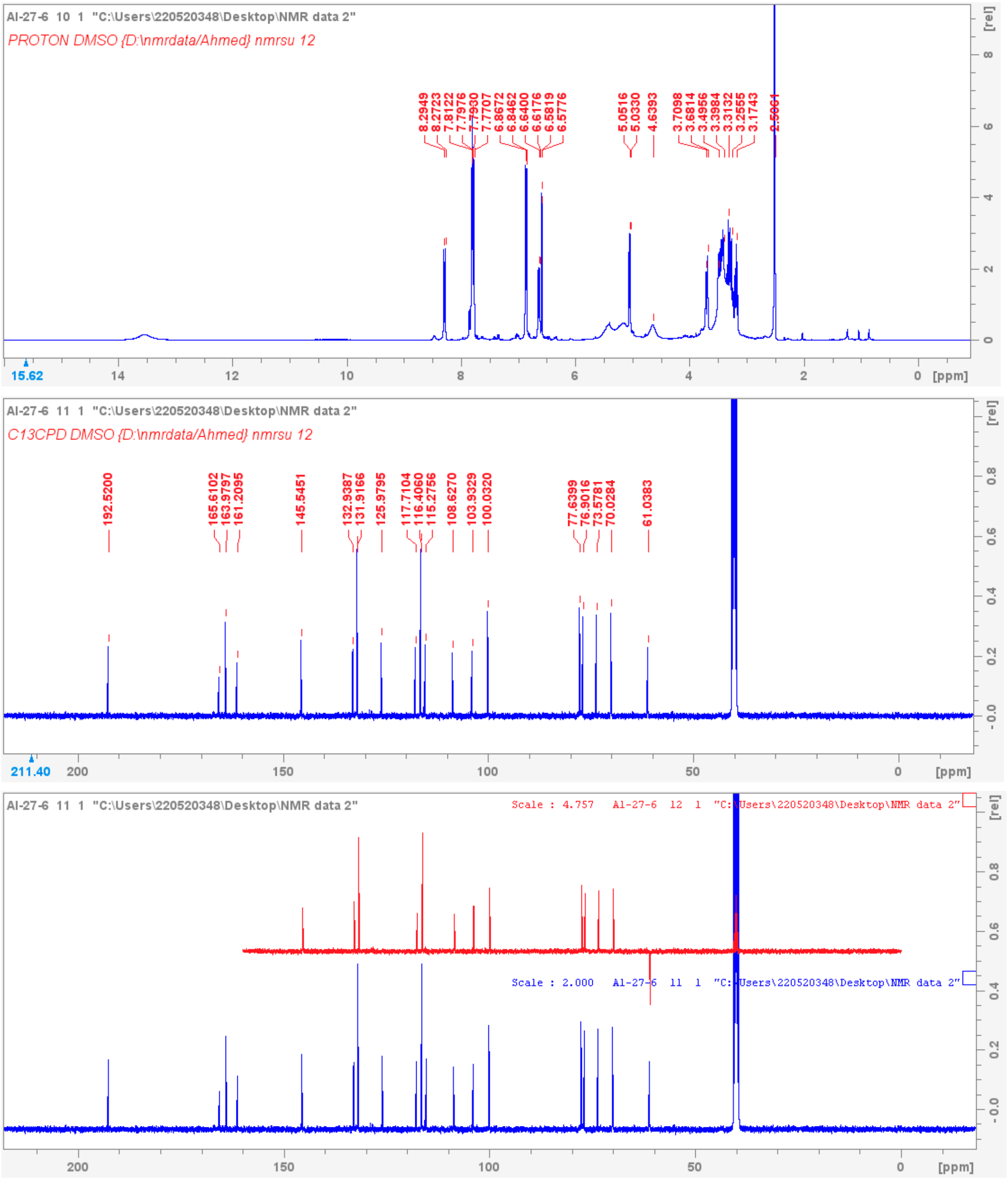

Figure S2.3. ^{1}H and ^{13}C spectra of compound **3.**

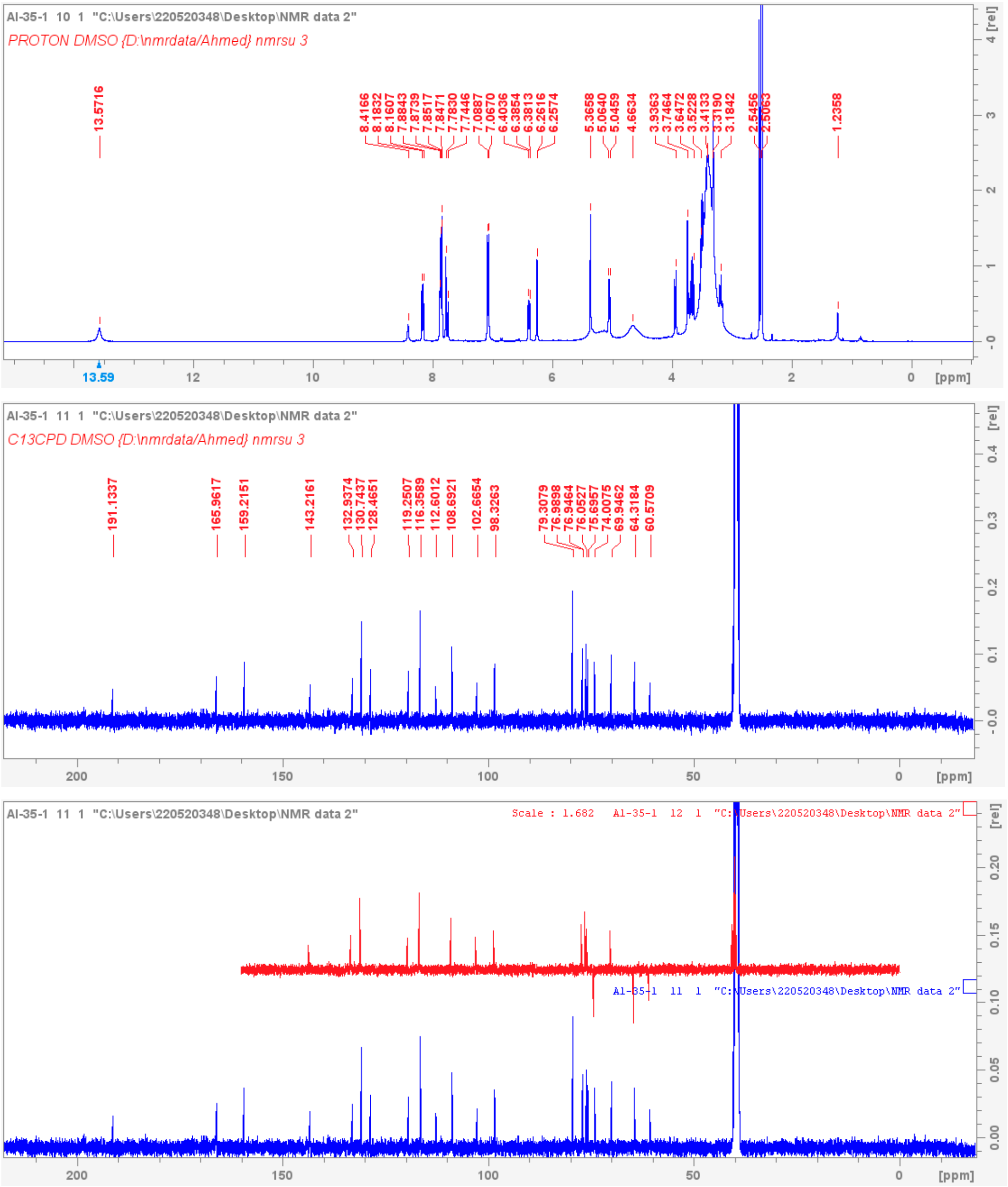

Figure S2.4. ^{1}H and ^{13}C spectra of compound **4**.

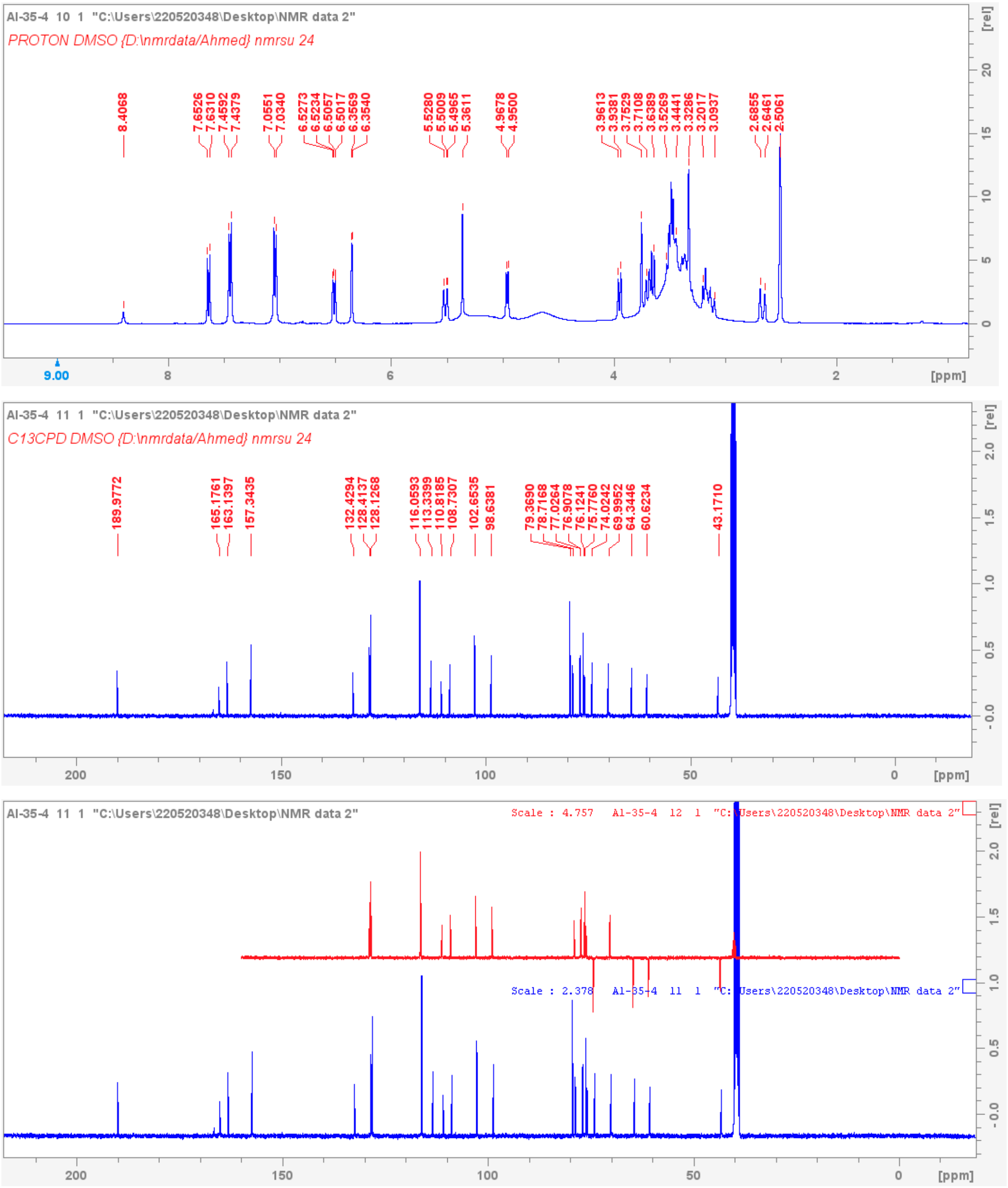

Figure S2.5. ^{1}H and ^{13}C spectra of compound **5**.

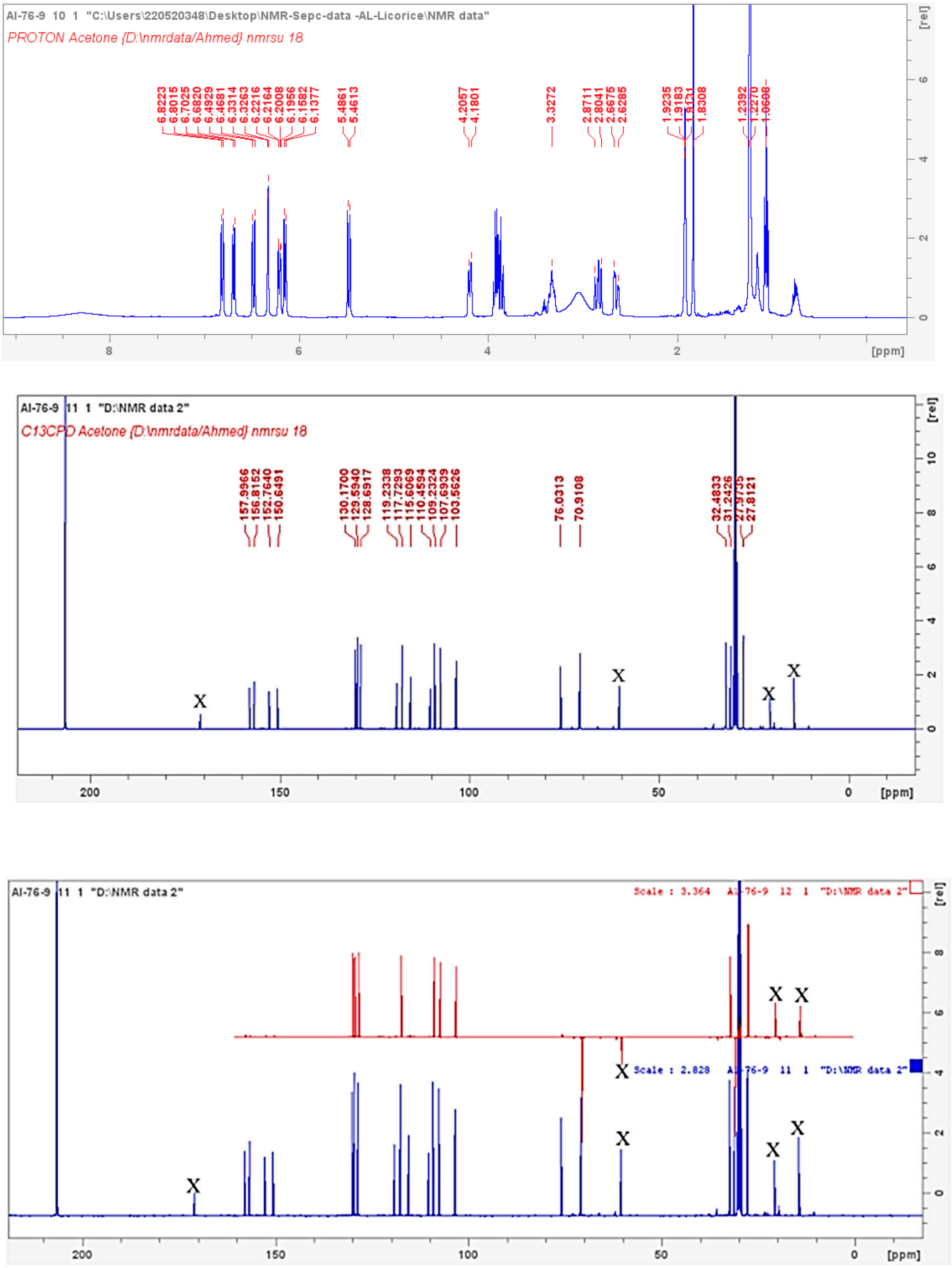

Figure S2.6. ^{1}H and ^{13}C spectra of compound **6** (the extra signals belong to traces of ethyl acetate).

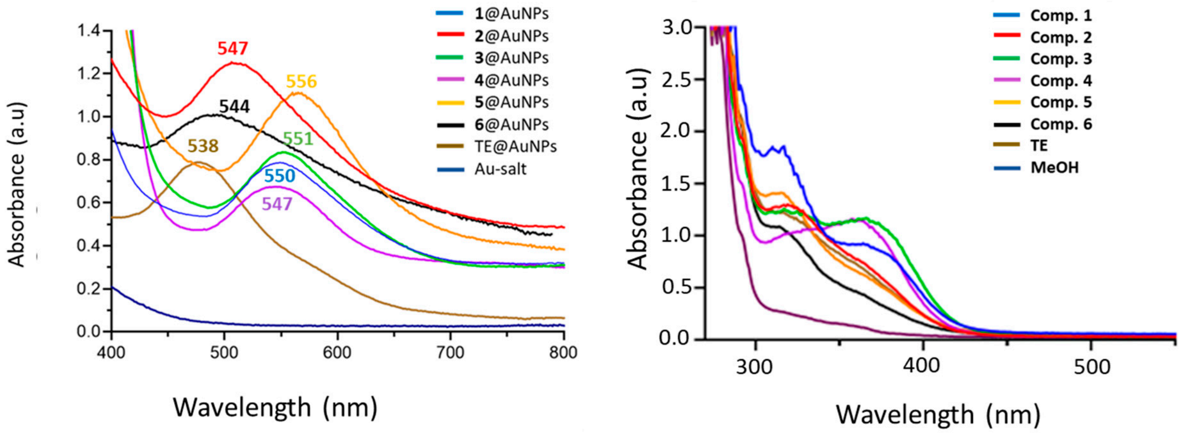

Figure S3. Ultraviolet-visible spectra of the green synthesized AuNP conjugates and the intact extract/pure compounds.

NPs	Zeta potential	Average Size/PDI
1@AuNPs	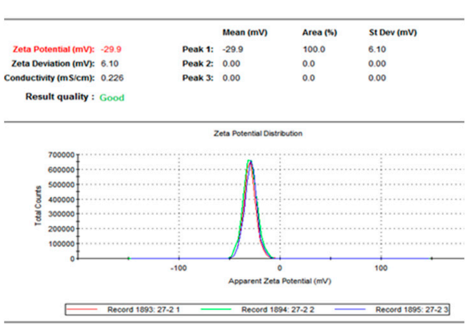	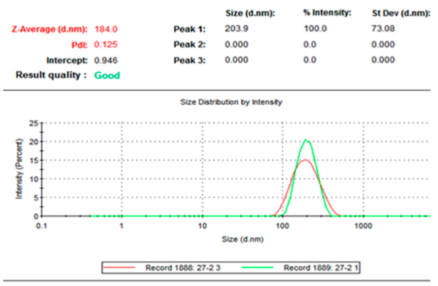
2@AuNPs	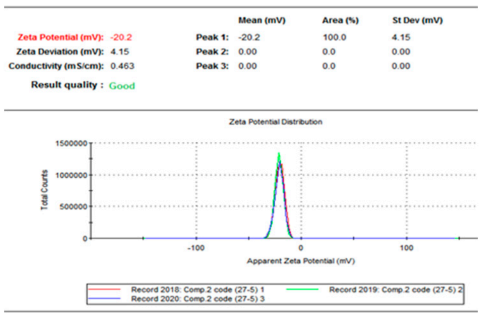	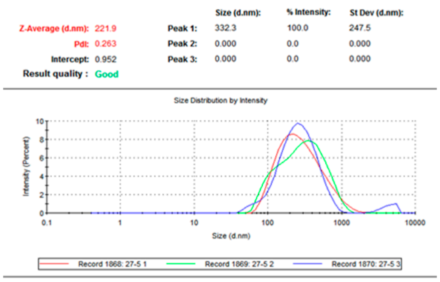
3@AuNPs	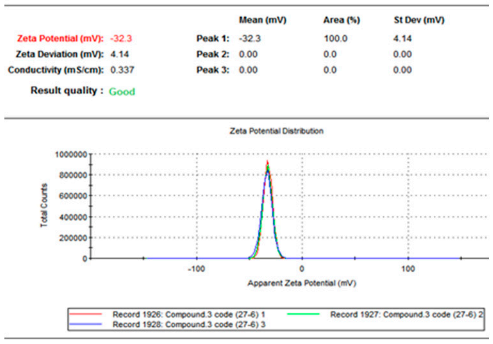	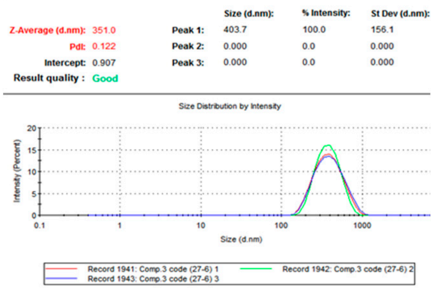

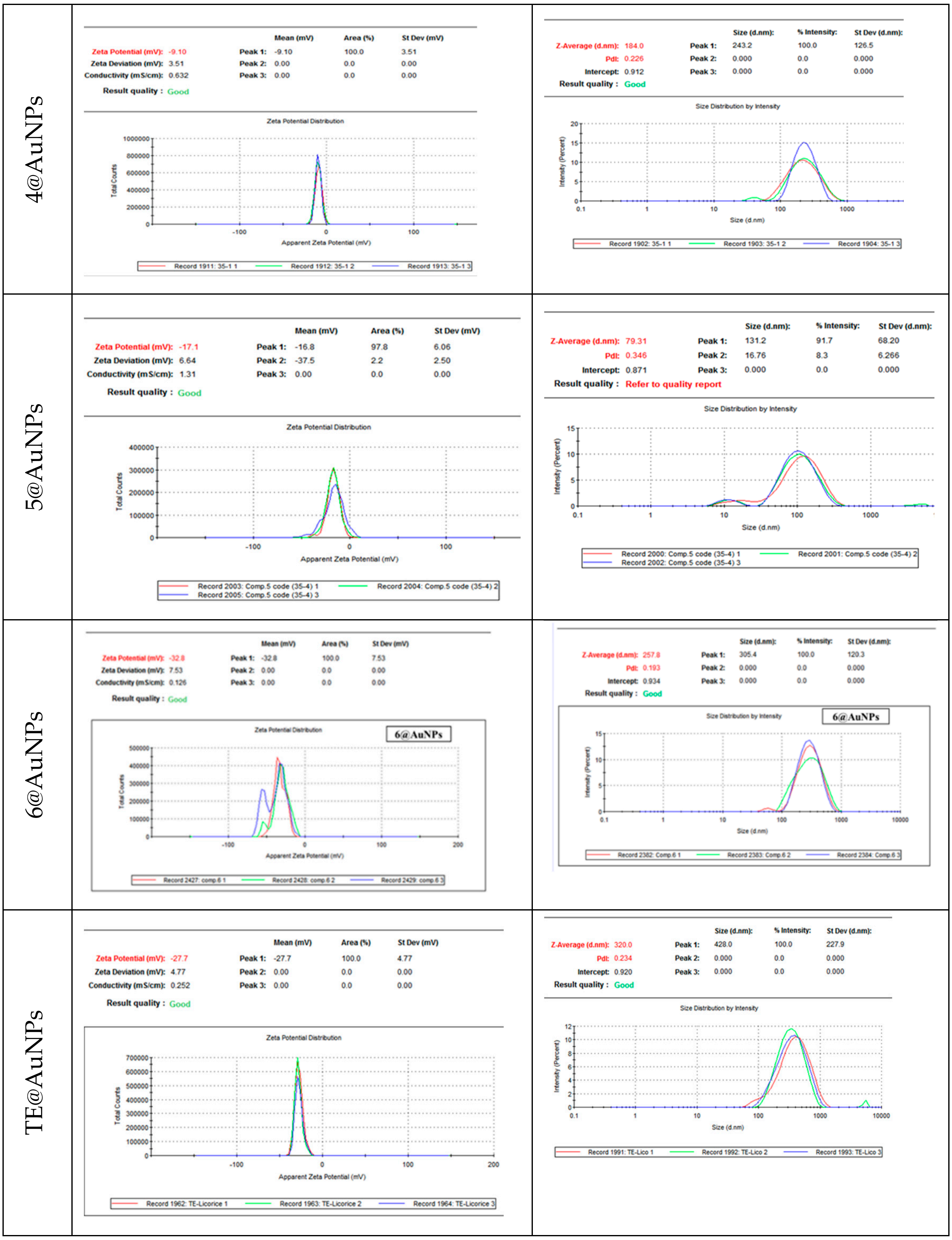

Figure S4. Zeta potential and relative size distribution of the synthesized NPs.

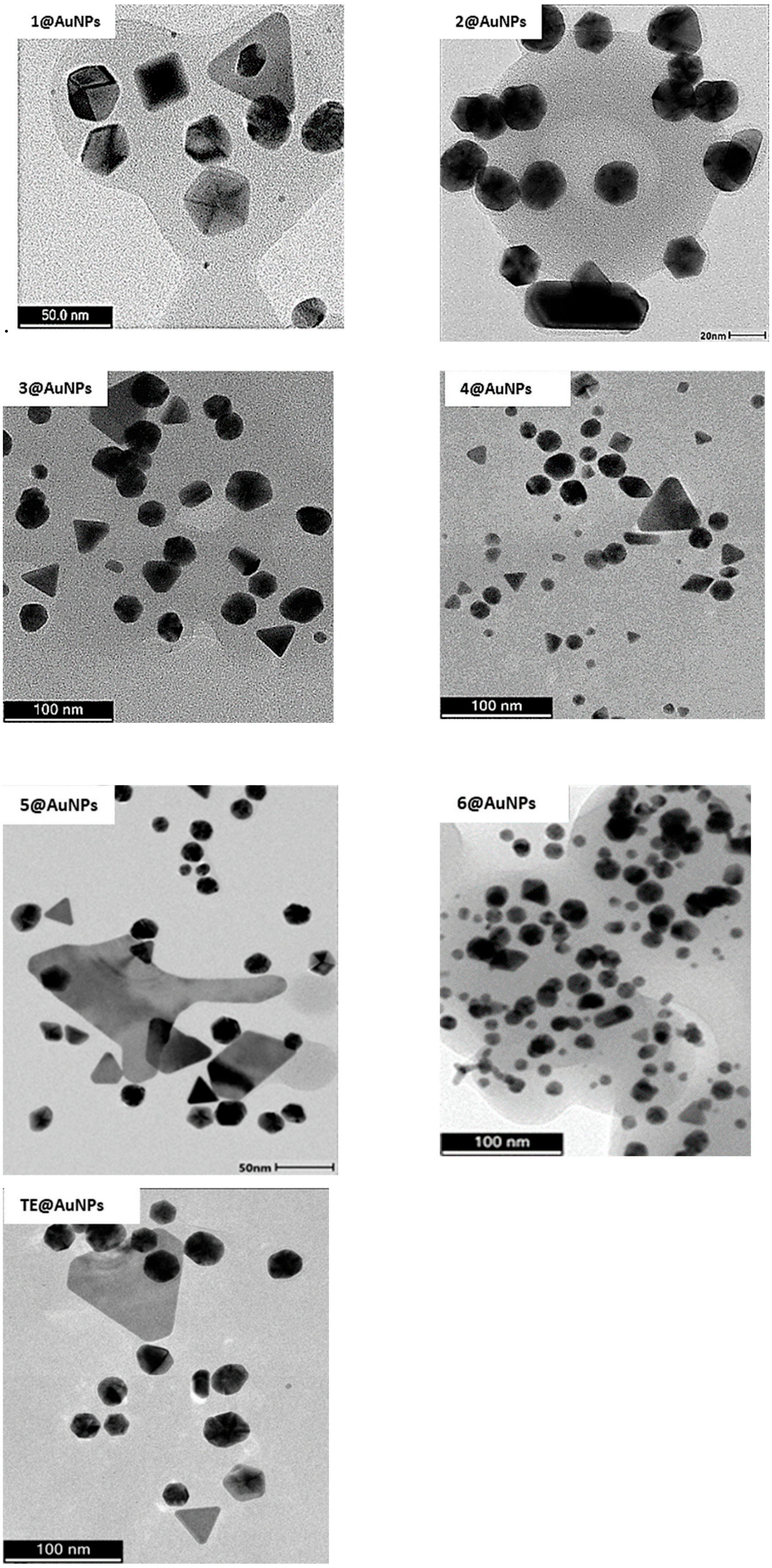

Figure S5. HRTEM of the synthesized AuNPs.

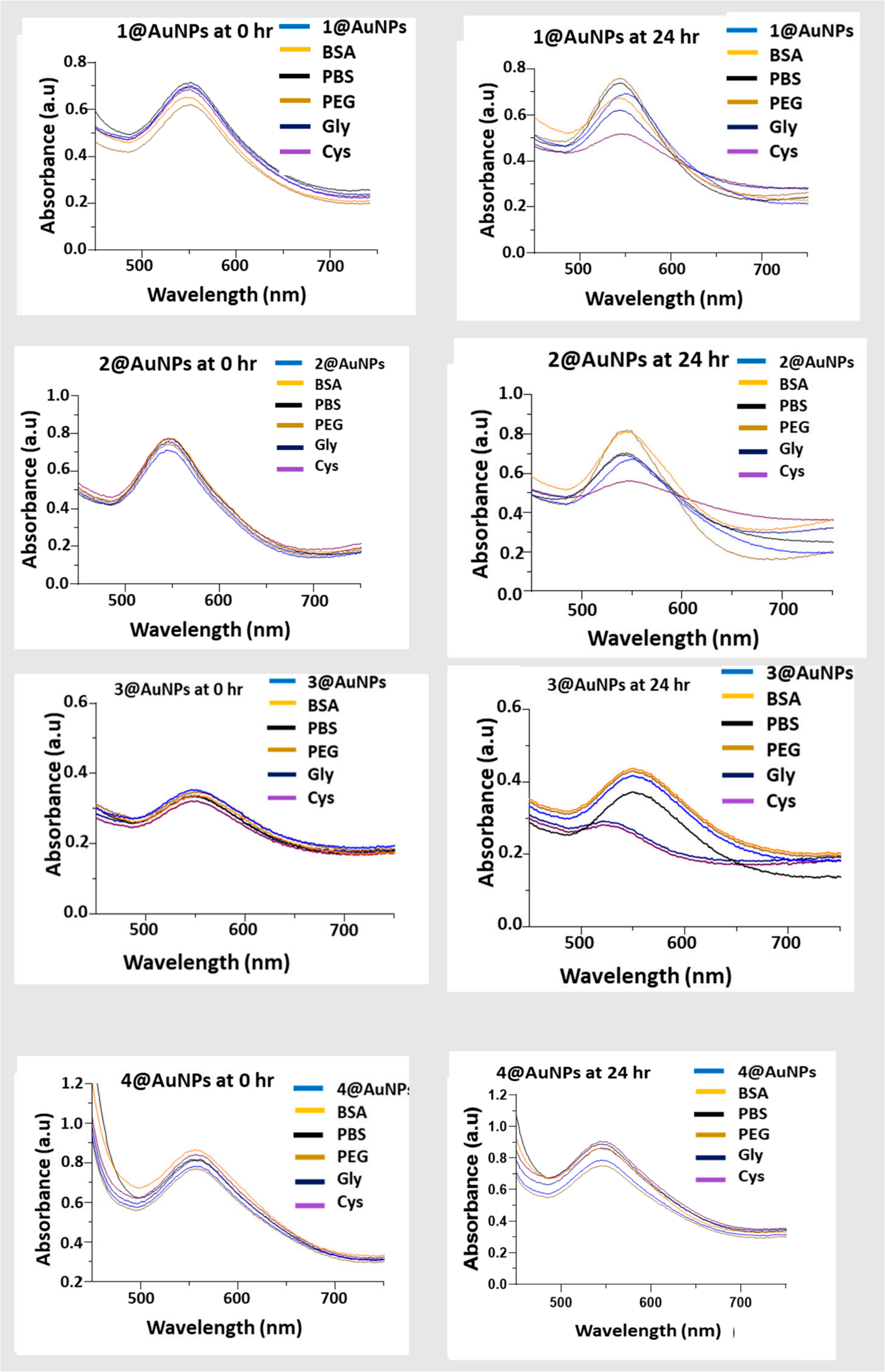
1@AuNPs at 0 hr
1@AuNPs
BSA
PBS
PEG
Gly
Cys
Absorbance (a.u)
Wavelength (nm)
1@AuNPs at 24 hr
2@AuNPs at 0 hr
2@AuNPs
2@AuNPs at 24 hr
3@AuNPs at 0 hr
3@AuNPs
3@AuNPs at 24 hr
4@AuNPs at 0 hr
4@AuNPs
4@AuNPs at 24 hr

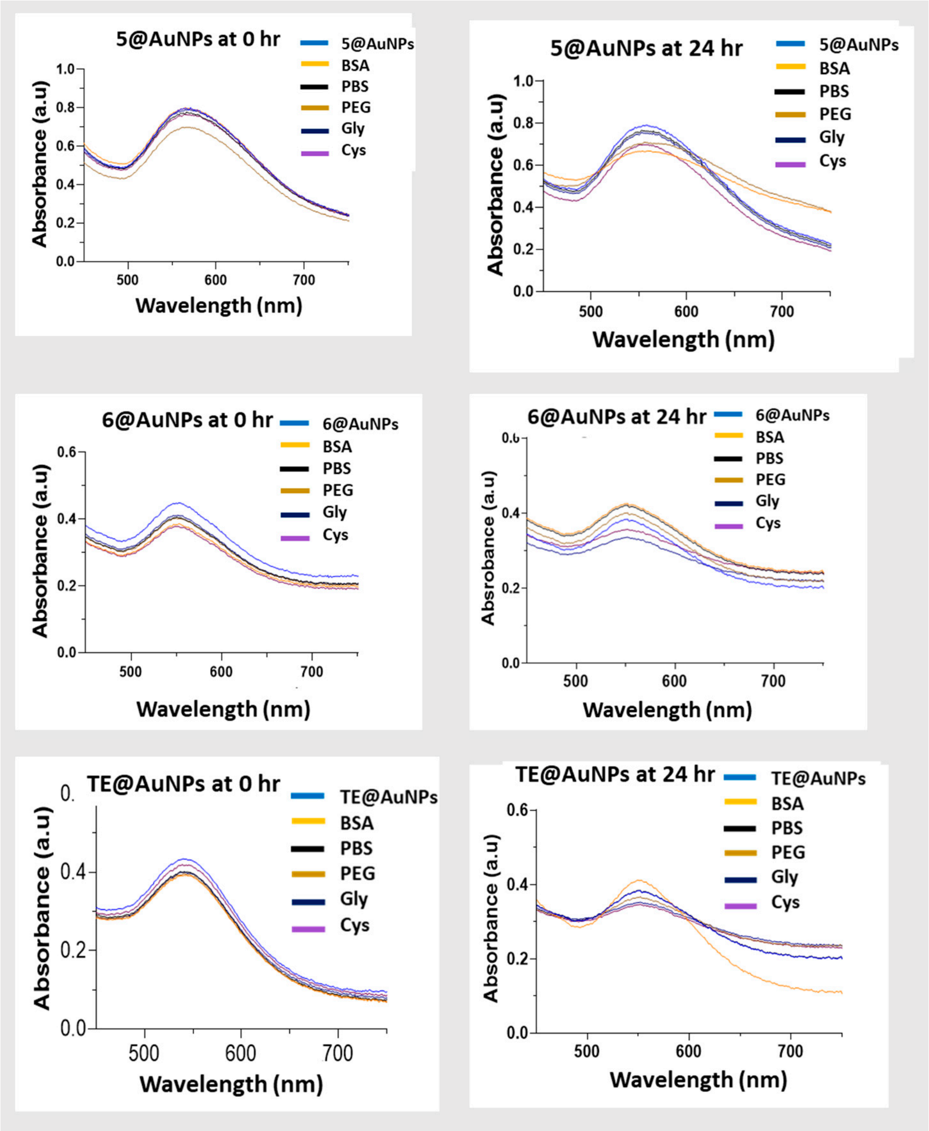

Figure S6. Stability of the AuNP conjugates in different biogenic media after 24 hr.

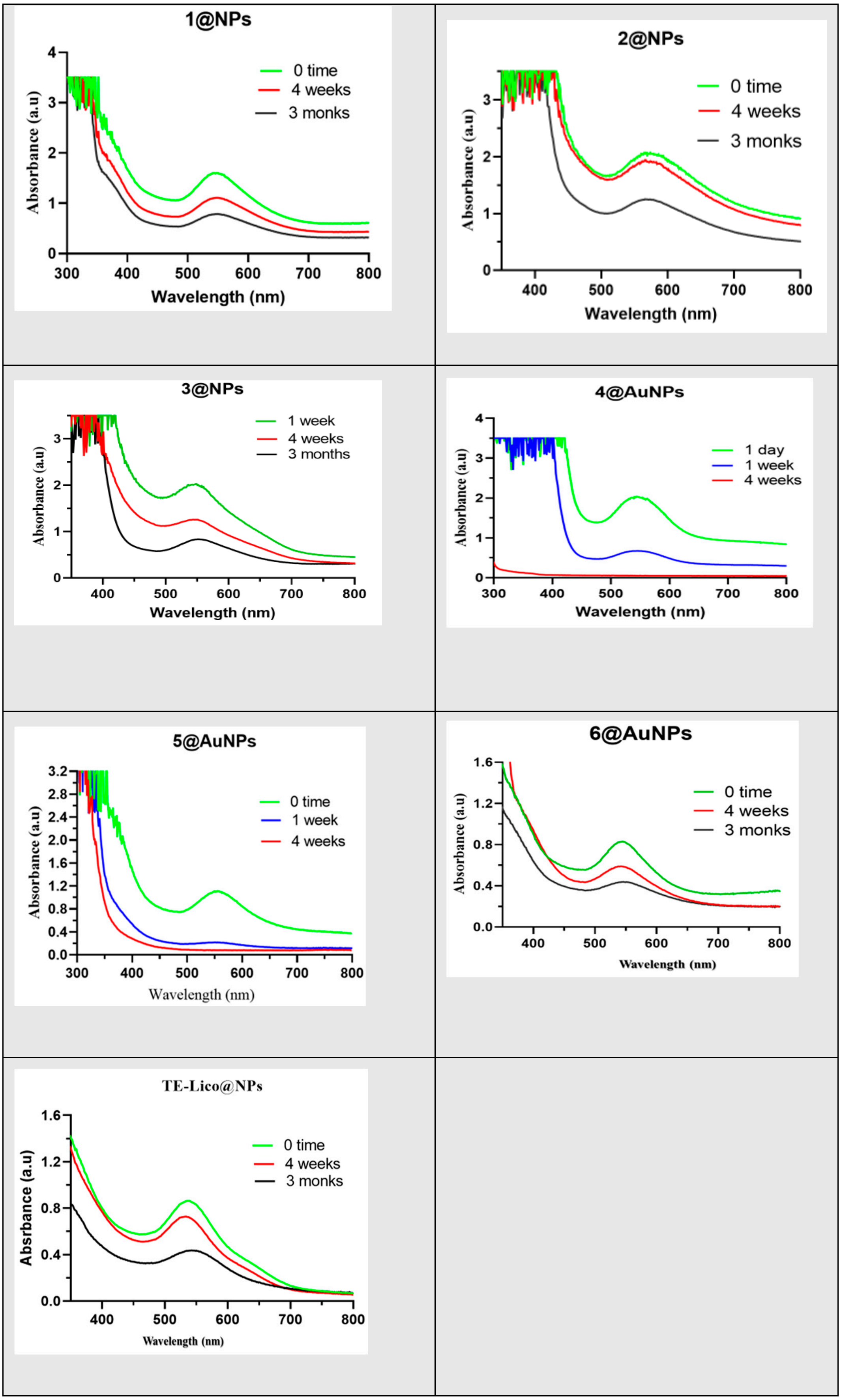

Figure S7. Stability of the AuNP conjugates for three months.

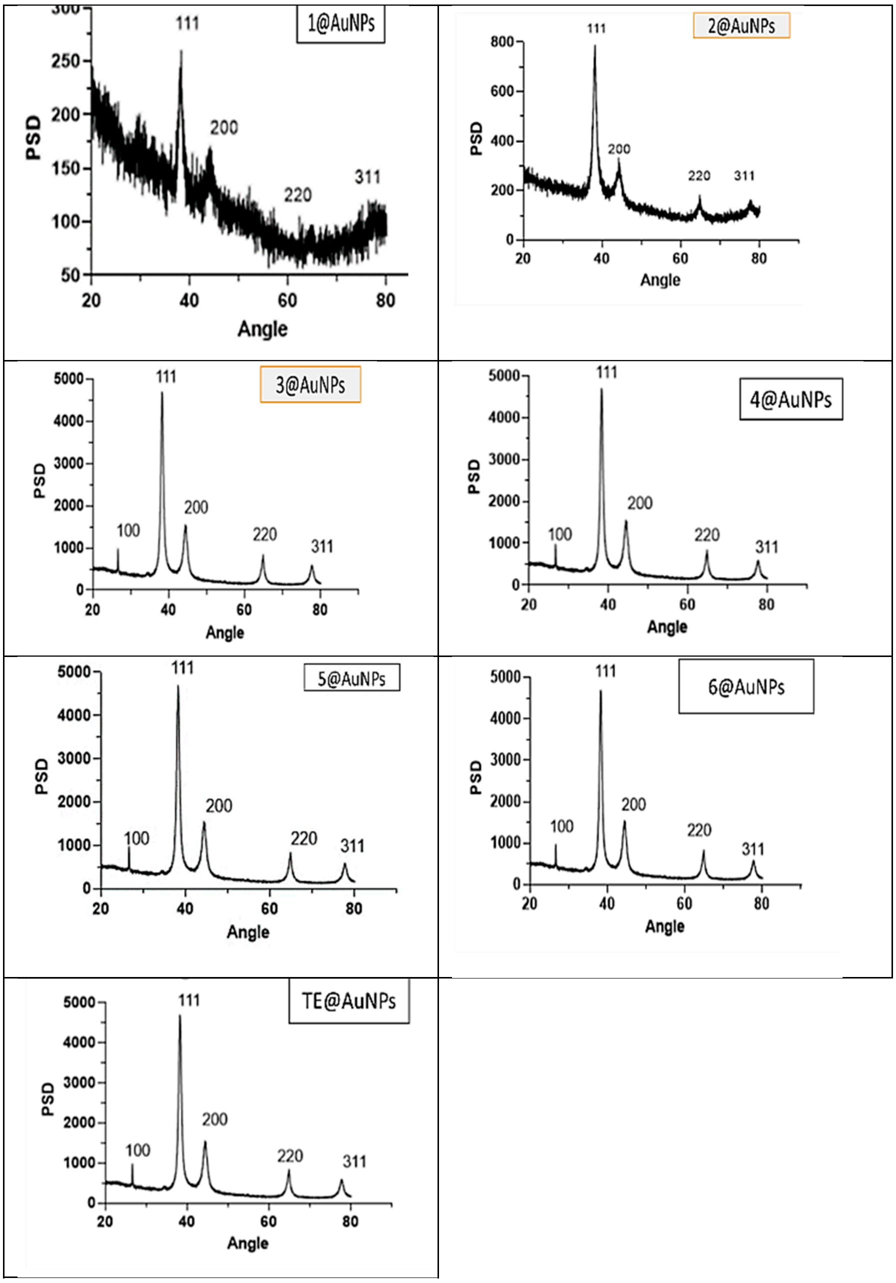

Figure S8. XRD of the synthesized AuNPs.

The average size of the particles (Table 1) was calculated using the Scherrer equation:

$$D = \frac{K\lambda}{\beta \ \mathrm{COS}\theta}$$

Where D = particle size, λ is the X-ray wavelength; β is the full width at half maximum (FWHM) of the diffraction peak; θ is the diffraction angle, and K is the Scherrer constant).

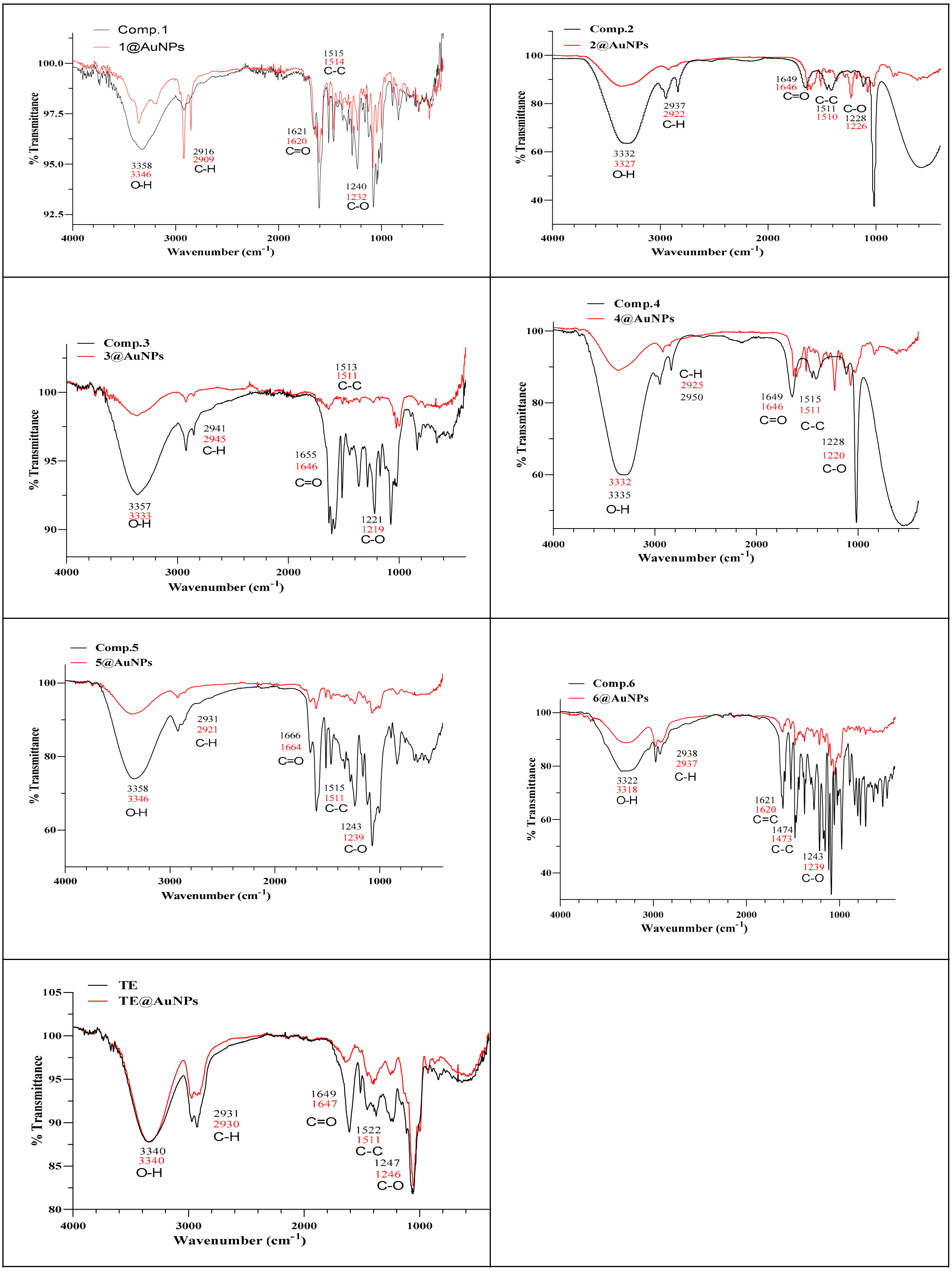

Figure S9. FTIR spectra of the synthesized AuNPs with their intact compounds.

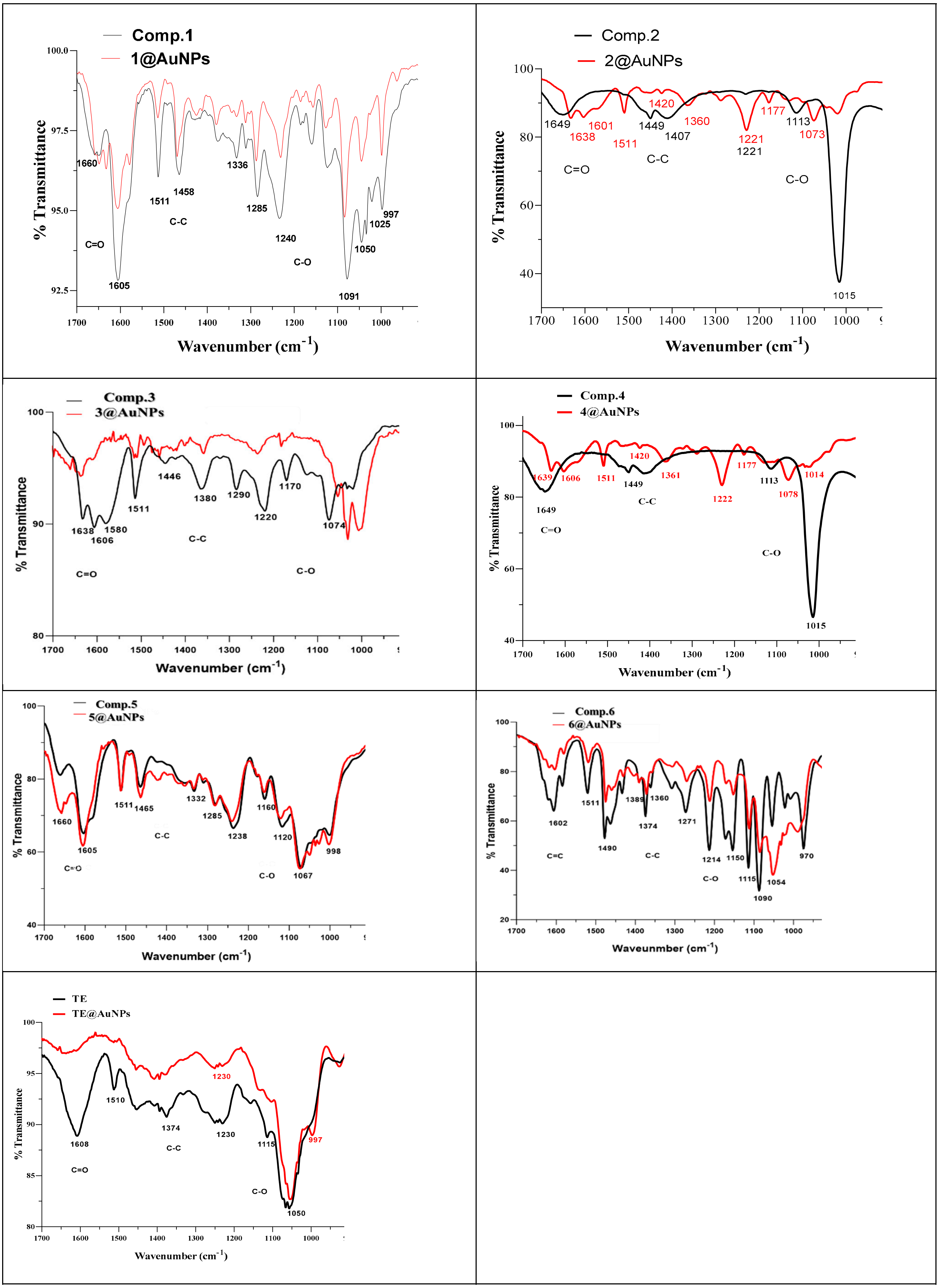
Comp.1
1@AuNPs
% Transmittance
Wavenumber (cm^{-1})
1660
1605
1511
1458
1336
1285
1240
1091
1050
1025
997
C=O
C-C
C-O
Comp.2
2@AuNPs
1649
1638
1601
1511
1449
1420
1407
1360
1221
1177
1113
1073
1015
C=O
C-C
C-O
Comp.3
3@AuNPs
1638
1606
1580
1511
1446
1380
1290
1220
1170
1074
C=O
C-C
C-O
Comp.4
4@AuNPs
1649
1639
1606
1511
1449
1420
1361
1222
1177
1113
1078
1014
1015
C=O
C-C
C-O
Comp.5
5@AuNPs
1660
1605
1511
1465
1332
1285
1238
1160
1120
1067
998
C=O
C-C
C-O
Comp.6
6@AuNPs
Waveunmber (cm^{-1})
1602
1511
1490
1389
1374
1360
1271
1214
1150
1115
1090
1054
970
C=C
C-C
C-O
TE
TE@AuNPs
1608
1510
1374
1230
1115
1050
997
C=O
C-C
C-O

Figure S10. FTIR of total extract/pure compounds (black) and their corresponding AuNPs (red) in the 1700-930 cm^{-1} range.

Table S1. The calculation using Scherrer equation*.

	Peak position	D (size nm)	Average size nm
1@AuNPs	38.09536	7.8765679	**6.564237061**
	44.1069	5.251906222	
2@AuNPs	38.15768	6.61590384	**5.486724806**
	44.18007	4.357545772	
3@AuNPs	38.25966	8.499486133	**10.01805687**
	44.36581	5.430383976	
	64.77982	12.48087356	
	77.70322	13.66148381	
4@AuNPs	38.25966	8.499486133	**9.248090944**
	44.3658	8.672004152	
	64.77982	9.509615469	
	77.70323	10.31125802	
5@AuNPs	38.25959	8.539102328	**10.05736089**
	44.36605	5.441996866	
	64.7802	12.54647926	
	77.70334	13.70186509	
6@AuNPs	38.25966	8.499486133	**10.01798554**
	44.3658	5.430280668	
	64.77982	12.48087356	
	77.70323	13.66130181	
TE@AuNPs	64.7802	12.54647926	10.05739385
	38.25959	8.539015613	
	44.36605	5.442031386	
	77.70334	13.70204914	

*the calculation was made using Origin software.
